# Observational reinforcement learning in children and young adults

**DOI:** 10.1038/s41539-024-00227-9

**Published:** 2024-03-13

**Authors:** Julia M. Rodriguez Buritica, Ben Eppinger, Hauke R. Heekeren, Eveline A. Crone, Anna C. K. van Duijvenvoorde

**Affiliations:** 1 Department of Psychology, University of Greifswald, Greifswald, Germany; 2 Berlin School of Mind and Brain & Department of Psychology, Humboldt University of Berlin, Berlin, Germany; 3 Department of Education and Psychology, Freie Universität Berlin, Berlin, Germany; 4 Department of Psychology, Concordia University, Montreal, Canada; 5 Department of Psychology, Technische Universität Dresden, Dresden, Germany; 6 Executive University Board, Universität Hamburg, Hamburg, Germany; 7 Department of Psychology, Education and Child Studies, Erasmus University Rotterdam, Rotterdam, Netherlands; 8 Institute of Psychology, Leiden University, Leiden, The Netherlands; 9 Leiden Institute for Brain and Cognition, Leiden, The Netherlands; 10 Present Address: Department of Education and Psychology, Freie Universität Berlin, Berlin, Germany

**Keywords:** Reward, Social neuroscience

## Abstract

Observational learning is essential for the acquisition of new behavior in educational practices and daily life and serves as an important mechanism for human cognitive and social-emotional development. However, we know little about its underlying neurocomputational mechanisms from a developmental perspective. In this study we used model-based fMRI to investigate differences in observational learning and individual learning between children and younger adults. Prediction errors (PE), the difference between experienced and predicted outcomes, related positively to striatal and ventral medial prefrontal cortex activation during individual learning and showed no age-related differences. PE-related activation during observational learning was more pronounced when outcomes were worse than predicted. Particularly, negative PE-coding in the dorsal medial prefrontal cortex was stronger in adults compared to children and was associated with improved observational learning in children and adults. The current findings pave the way to better understand observational learning challenges across development and educational settings.

## Introduction

Numerous findings indicated that others have a strong impact on learning and decision-making^1–6^. For instance, we may be swayed by the opinions of others to adjust our norms on acceptable behavior. Alternatively, others could be considered a source of information, as they can provide us with valuable information about our environment. Learning from observing others’s behaviors and outcomes may have benefits in dangerous or novel environments, in which observational learning allows us to learn from the actions and outcomes of others without having to engage in these (potentially hazardous) behaviors ourselves. Social situations lend themselves well for observational learning. That is, learning from others is ubiquitous on playgrounds, in schools and other social environments, in which we have the opportunity to observe others behaviors and subsequent outcomes without necessarily participating ourselves. However, compared to learning from own experiences, the developmental mechanisms underlying observational learning are poorly understood.

Several studies have examined how learning from own outcomes changes with age^7,8^. Many studies observed that adults typically outperform children during instrumental learning. That is, adults learn faster than younger ages and choose the most rewarding option more often. This is thought to be related to a developmental improvement in cognitive control, including behaviors such as sustained attention and working memory, which would benefit learning. It is yet unclear how a social observational context influences this adult advantage. That is, children are shown to be highly sensitive to the example of others, quickly copying behavior, particulary of their own peers^9^. On the other hand, a previous observational learning study showed that children may not process and use information of others as efficiently as young adults in their learning and decision making^10^. These age-related differences in observational learning have been related to differences in temporal processing of observed outcomes using electro-encephalogram (EEG)^9,10^. Children, ages 8–10, showed larger electrophysiological responses when observing peers as compared to adults^5^, but compared to adults their electrophysiological responses did not change according to their learning^6^, and they could not yet benefit in accuracy from observed information as much as adults. What remains unresolved in the current literature are the functional and computational processes supporting observational learning as compared to individual learning across development. In this study, we therefore applied a model-based neuroimaging approach to observational learning across development, by combining reinforcement learning (RL) modeling and functional magnetic resonance imaging (fMRI) in a children’s and adult age group.

Computational models of reinforcement learning haves been successfully applied to understand reinforcement learning in adults and children^5,11–18^. In these models, learning is driven by prediction errors, which reflect the mismatch between expected, Q_a_(t), and received outcomes, r(t), per trial (t). Whenever an outcome is better (worse) than expected, the model will generate a positive (negative) prediction error. Prediction errors are thought of as important learning signals, and are shown to scale with activity of midbrain dopamine neurons^19–22^. The weight in which prediction errors drive learning behavior is quantified by learning rates. High learning rates allow prediction errors to quickly change the value of choice options. Low learning rates result in slower updating and therefore a long-term integration of outcomes in the value of choice options. In addition, RL models typically include a temperature parameter that specifies how precisely one’s choices discriminate between the value of choice options, and thus controls the specificity in choice behavior. Previous research involving reinforcement learning models has yielded mixed results regarding age-related differences in positive and negative learning rates^8^. Recent reviews, however, indicate that choice specificity tends to consistently increase with advancing age^7,8^.

In contrast to individual learning situations, observational learning is a richer learning environment. For instance, behavior can be updated twice: First, by an experiential prediction-error (as in standard reinforcement learning) and second by an observational prediction-error. Where the first is generated by one’s own outcomes, the latter is generated by the outcomes of an observed other. Neuroimaging studies examining the underlying neural mechanisms have suggested neural systems may be partly overlapping and partly specific for learning from social and non-social outcomes^5,16,23,24^. That is, whereas prediction errors in various learning paradigms have been represented in brain regions such as the ventral striatum and ventral medial prefrontal cortex (vmPFC)^11,25–27^, prediction errors in other brain regions may be more specialized for social learning. For instance, social learning has been related specifically to the dorsal medial PFC (dmPFC)^28,29^ and anterior cingulate cortex (ACC)^11,12^ with the latter representing other-related reward-processing, and information on the consequences of actions of others^24,30^. In addition, social learning has been related to brain regions involved in mentalizing and modeling of others, such as the dmPFC, temporal-parietal junction (TPJ), and posterior superior temporal sulcus (pSTS)^23^. Recent studies highlighted the role of mentalizing particularly in situations in which individuals are confronted with opposing preferences and in which the mental state (goals, preferences, beliefs, or intentions) of another person needs to be inferred^14,28,31^. This mentalizing network is also thought to track trial-level updating during strategic social interactions^12,32^.

Until now, few studies have examined the computational and neural correlates of age-related differences in social and non-social reinforcement learning. We build on this previous work and extend this with the current study: Here, we aim to examine age-related differences in the neural correlates of observational and individual prediction errors. Including both observational and individual learning allows us to examine age-related differences in both learning situations, as well as to compare these learning situations directly. To do so, we used functional magnetic resonance imaging (fMRI) and a computational modeling approach. Thirty children (8–10-year-olds, 18 female) and 30 young adults (18–20-year-olds, 16 female) participated in this study. For the youngest ages we focused on middle childhood, including children between 8 and 10 years. In this way we captured an important phase of age-related differences in controlling responses towards positive and negative feedback^33^, and we built on previous EEG work on observational learning^9,10^.

**Fig. 1:**
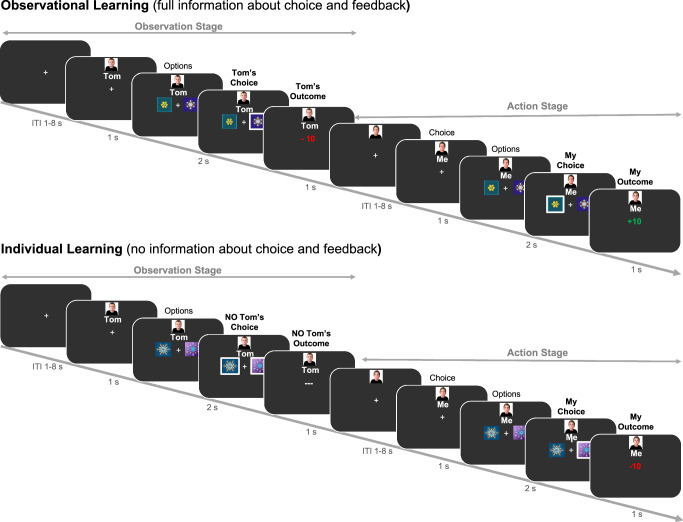
Experimental design. Example of the trial procedure for the observation learning and individual learning condition.

**Fig. 2:**
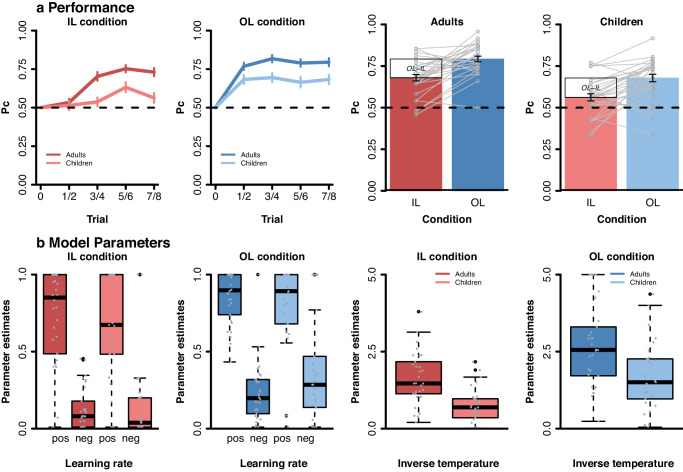
Performance and computational parameter differences in individual and observational learning. **a** Proportion correct choice (Pc). Pc displayed separately for the two age groups (adults, children) and learning conditions (individual (IL), observational (OL)). Data were averaged into four bins (across eight trials). Error bars reflect the SEM. Grey lines reflect individual learning differences between IL and OL condition. **b** Parameter estimates of the best fitting model across age groups. Learning rate (i.e., alpha pos and alpha neg) and inverse temperature (i.e., beta) per age group and condition (individual learning (IL) and observational learning (OL)).

Before the start of the experiment, participants met an age-matched and same-sex peer who was introduced as the observed other player in the task. Participants performed a probabilistic observational learning paradigm^9–11^ (see Fig. 1). In this task, participants made repeated choices between pairs (i.e., 8 in total per pair) of stimuli, where one stimulus was associated with a high probability of reward (80% gains, 20% losses) and the other was associated with a low probability of reward (20% gains, 80% losses). Before participants could choose, they observed the other player choosing between the same two stimuli. We manipulated the amount of observable information of the other player across two learning conditions with each three runs (each with 2 stimulus pairs per learning condition; thus 32 trials per run): In the *individual learning condition* participants received no information about the behavior of the other individual; in the *observational learning condition* they could observe the other player’s actions and outcomes before making their own choice. With this setup, we can better understand how observed information of others is used for one’s own learning. The four stimulus pairs (two per condition) per run were presented intermixed. To better understand and explain age-related differences in learning in this task, we used a double update Q-learning algorithm^11^ that captures learning from both other’s and own outcomes. Note that just in the observational learning condition, Q-values could be updated in the observational stage as in the individual learning no outcome was displayed (see Fig. 1; and Methods). Here, we included two independent learning rates (α_pos_, α_neg_), separately for each valence (positive, negative) of the outcome for both learning conditions. A higher learning rate means a quicker updating of expected values and thereby faster learning. For both stages and conditions, the probability of choosing option *a* from a stimulus pair (ab) was computed using an inverse softmax function^34^ (see Methods for further details), were we estimate the inverse temperature (*β*)) indicating the specificity of the subject to differences in Q-values. Since *β* is the *inverse* temperature, this means that higher values indicate a less deviations from optimal choice behavior (i.e., a greater choice specificity). For the fMRI analyses we used trial-level calculated prediction errors when presented with own (action phase in IL condition; Fig. 1) and others (observational phase in OL condition; Fig. 1) outcomes derived from the median of the parameter estimates (i.e., α_pos_, α_neg_ and *β*) per age group (Fig. 2). Using a model-based parametric fMRI approach, we examined which brain regions correlated with prediction error activation.

Given our previous developmental work, we expected that both age-groups benefitted from the additional observational information, although adults are expected to outperform children in both conditions and are expected to learn faster than children^10^. Based on work in adults, we expect that RL-models can be used to describe individual and observational learning across development, although during observational learning choice values will be updated twice^11^. Given previous mixed findings on age-related differences in learning rates, we had no clear predictions here, but expected that the inverse temperature parameter is sensitive to age-related differences^8^. Based on previous developmental work, prediction-error activation of own and observed outcomes is expected to be related to striatal and medial prefrontal cortex activation across development^9,10^. Observational learning is expected to relate to the medial PFC including the ACC and dmPFC^5,11,12,23,24,30^, and mentalizing regions such as the TPJ^14,28,31^. Finally, whereas both age groups may benefit from observational compared to individual learning, we expected greater neural differentiation between own and observed outcomes in adults compared to children^10,35^, reflective of greater efficiency of learning which is increased in observational situations. Taken together, we include a computational approach to examine the neural associations and age-related differences in observational versus individual learning between children and adults.

## Results

### Performance differences in observational and individual learning

One participant (child) did not finish the learning session in the scanner and was not included in further analyses (see Methods). Both age groups selected the correct option (rewarded 80% of the time) more frequently than the incorrect option (rewarded 20% of the time) and showed accuracies above chance level in both learning conditions (four *t-*tests against chance level per condition and per age group; all *p*’s <0.001). Performance was correlated between the observational and individual learning condition (Persons’ *r* = 0.46, *p* < 0.001), indicating that individuals who performed well in the observational condition, also performed well in the individual learning condition. Descriptively, 47 (24 children and 23 adults) out of 59 subjects performed more accurately in the observational than the individual condition, suggesting that most participants benefitted from the additional observable information.

To test age group and condition effects on learning we analyzed choice behavior (averaged across runs) using a mixed-effects general linear model (controlling for intelligence) with the between subject predictor age group (children, adults) and the within subject predictors learning condition (individual, observational) and trial number (1:8), as well as all interaction terms (see Methods). The results of this analysis (see Supplementary Table 1 for full model output and Fig. 2a for visualization) showed significant main effects of learning condition (β = 0.12, *t* = 5.2, *p* < 0.001) and trial number (β = 0.04, *t* = 3.0, *p* = 0.005), as well as a significant learning condition x trial number interaction (β = −0.04, *t* = -2.2, *p* = 0.026). Post-hoc comparisons per condition revealed that participants improved across trials in the individual learning condition (β = 0.06, *t* = 6.3, *p* < 0.001), while performance remained stable in the observational condition (see Fig. 2a; β = 0.004, *t* = 0.43, *p* = 0.671). This suggests that learning from observing others resulted in relatively high-performance levels early in the learning process, while individual learning showed a more gradual improvement across trials. We also found age-related differences: including a significant main effect of age group (β = 0.1, *t* = 3.7, *p* < 0.001), as well as a significant age group x trial number interaction, β = 0.04, *t* = 2.2, *p* = 0.031. Post-hoc comparisons per age group revealed that adults improved across trials (β = 0.05, *t* = 4.6, *p* < 0.001), while children showed limited improvement (see Fig. 2a; β = 0.18, *t* = 1.75, *p* = 0.08). This indicates that adults generally demonstrated greater accuracy improvement across trials compared to children. No significant age by learning condition interactions was observed (*p* = .9). Thus, altough adults overall outperformed children, the age groups benefitted to a similar degree from the additional observable information (see Fig. 2a).

To ensure that differences in learning conditions were not solely due to varying information levels provided to participants (i.e., more information in observational than individual learning condition), we conducted an additional mixed-model analysis. This analysis focused on an equal amount of information in each learning condition, comparing trials 1–4 in the observational learning condition (including 1/3/5/7 observed/received outcomes) to trial 2,4,6, and 8 in the individual learning condition (including 1/3/5/7 received outcomes). The results align with our main analysis, indicating better performance in the observational learning condition compared to the individual learning condition (main effect condition: β = 0.06, t = 5.03, *p* < 0.001), across both age groups (age x condition interaction: *p* = 0.8).

#### Computational parameter differences in individual and observational learning

Next, we examined age and learning condition effects on computational parameters (i.e., α_pos_, α_neg_ and *β*; see Methods). Higher learning rates indicate that recent choice outcomes have a stronger effect on future choices than less recent choice outcomes. The inverse temperature indicates participants’ sensitivity to differences in these choice values. Here, the higher the beta parameter, the less stochastic choice behavior was.

Robust mixed-effects analyses were used to test main and interaction effects of age group, condition (IL, OL), and valence (Pos, Neg; only for learning rates; see Fig. 2b and Supplementary Table 2). Findings showed that positive and negative learning rates differed significantly across learning conditions (β = 0.75, *t* = 3.4, *p* < 0.001, main effect of valence) with both learning rates were higher for positive (median = 0.78) than negative outcomes (median = 0.2). No significant main effect of condition, age, or any interactions with age and condition were observed (all *p’*s > 0.7). A similar analysis on the inverse temperature parameter (see Fig. 2b and Supplementary Table 3) showed that participants followed choice values more optimally in the observational (median = 2.14) than individual learning (median = 1.03) condition (main effect condition, β = 0.95, *t* = 7.4, *p* < 0.001). A main effect of age group showed that children (median = 1.13) were less optimal in their choice behavior than adults (median = 2.18; β = 0.91, *t* = 4.5, *p* < 0.001). No age x condition effect was observed (*p* = 0.9).

### Age group and condition differences in prediction-error activation

**Fig. 3:**
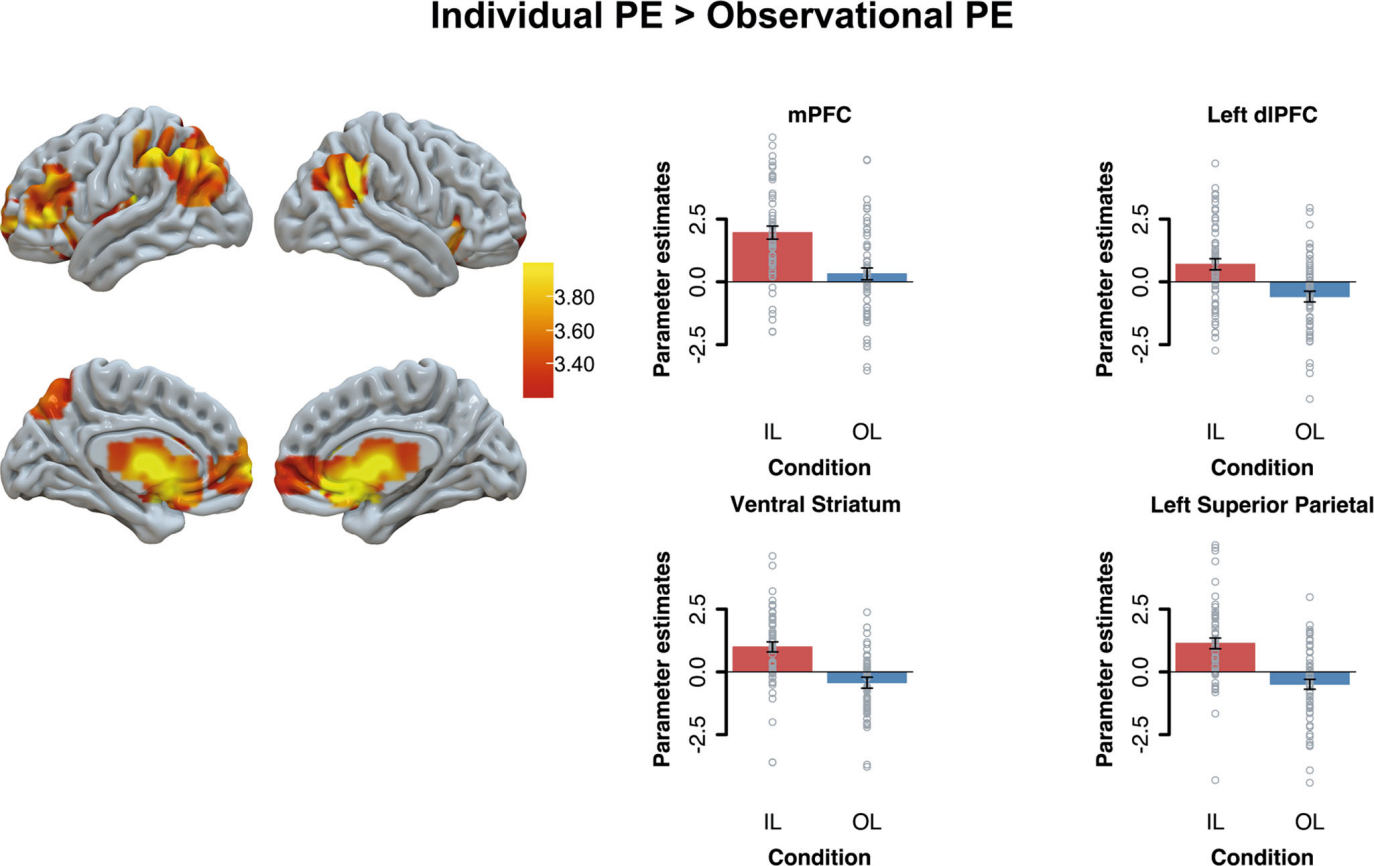
Activation clusters whole-brain effects for individual PE > observational PE. Results are displayed at Family-Wise Error (FWE) cluster-corrected *p* < 0.05, with an initial cluster forming threshold of *p* < 0.001. For visualization we extracted the beta-values from the whole-brain condition effect from regions of interest. Since our activation spanned multiple subcortical anatomical regions, the functional activation from the subcortical cluster is overlaid with a nucleus accumbens (ventral striatum) anatomical mask.

We analyzed neural activation related to prediction error coding (entered as a signed continuous trial-by-trial predictor) when participants received their own outcomes (Fig. 1, individual learning condition action phase) and others’ outcomes (Fig. 1, observational learning condition observational phase). We initially examined whether there were brain regions that responded differently to prediction errors in individual learning and observational learning conditions. Results showed that individual compared to observational prediction errors were more strongly related to activation in the vmPFC (*p*FWE’s < 0.05), the left lateral PFC (*p*FWE’s < 0.05), the bilateral striatum, and bilateral parietal cortex (*p*FWE’s < 0.001; see Fig. 3; and Supplementary Table 4). No brain regions correlated stronger to observational than individual prediction errors (all *p*FWE’s > 0.05).

**Fig. 4:**
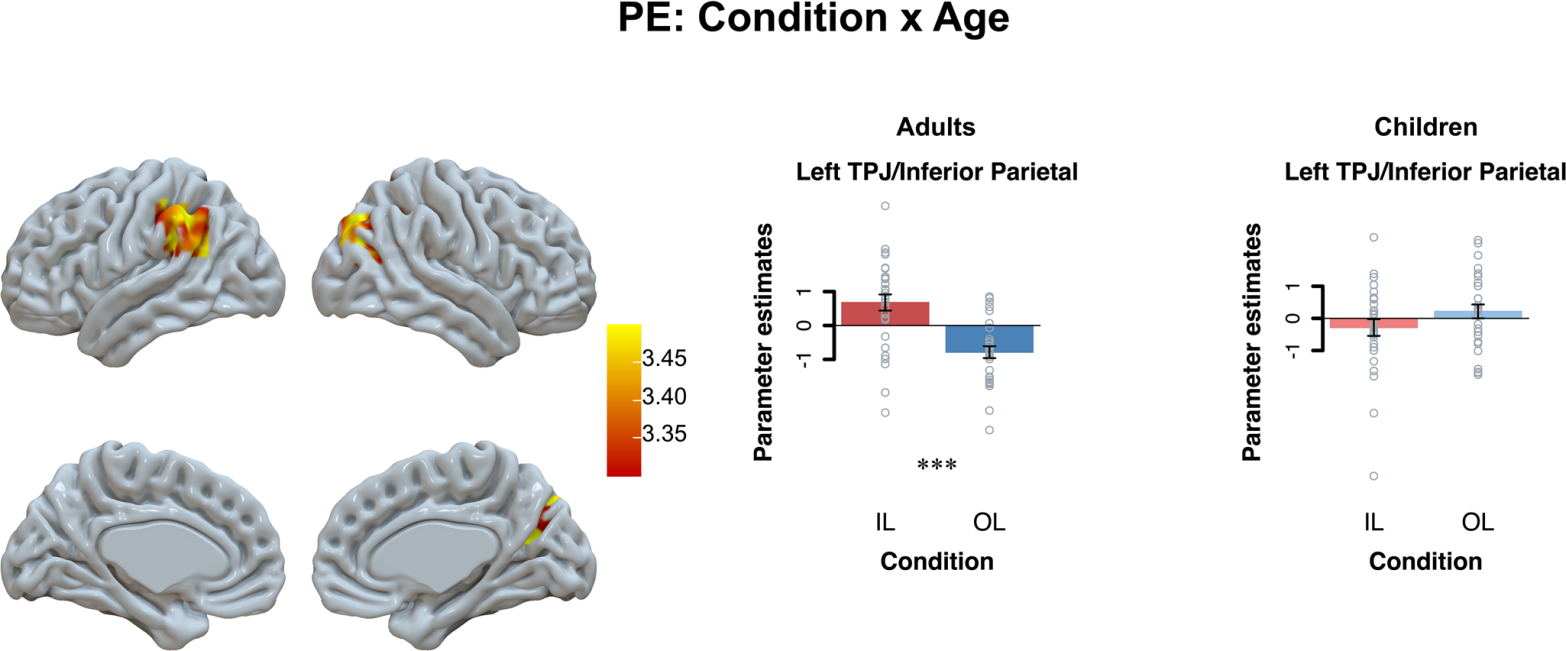
Activation clusters whole-brain analyses on PE-related activation: learning condition (IL > OL) × age (adults > children). Results are displayed at Family-Wise Error (FWE) cluster-corrected *p* < 0.05, with an initial cluster forming threshold of *p* < 0.001. For visualization we extracted the beta-values from the whole brain interaction effect of learning condition × age. Significant differences of follow-up tests are marked here (***p < 0.001).

Next, we examined with a whole-brain ANOVA whether this difference in prediction error activation between individual and observational learning conditions was sensitivity to age group differences. An age group × learning condition interaction was observed in the left TPJ/inferior parietal cortex (*p*FWE < 0.05; learning condition (IL > OL) × age (adults > children) contrast). Follow-up tests per age group showed that the TPJ/ inferior parietal cortex differently responded to individual and observational prediction errors in adults compared to children: That is, prediction errors related to increased TPJ activation when other’s outcomes were worse than expected and to increased activation when own outcomes were better than expected in adults (*t*(29) = 5.07, *p* < 0.001). This differentiation was not observed in in children (*p* = 0.15; see Fig. 4; and Supplementary Table 5).

#### Age effects in observational and individual prediction error activation

**Fig. 5:**
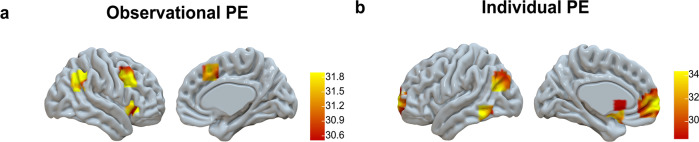
Activation clusters for PEs relative to (implicit) baseline. **a** observational PE activation and **b** individual PE activation, displayed based on a whole-brain *F*-test (pFWE <0.05) Observational PE activation showed negative correlations with brain activation (i.e., greater negative PEs resulted in larger activation, see bar charts of extracted beta-values Supplementary Fig. 1). Individual PE activation showed positive correlations with brain activation (i.e., greater positive PEs resulted in larger activation, see bar charts of extracted beta-values Supplementary Fig. 1).

We first used a whole-brain *F*-test (*p*FWE < 0.05). to examine both positive correlations with prediction error activation (i.e., regions where activation increased with larger positive prediction errors) and negative correlations with prediction error activation (i.e., regions where activation increased with larger negative prediction errors) within each learning condition. The results revealed that in the observational learning condition, prediction errors were associated with activation in the right lateral PFC, right inferior parietal, and right insula (see Fig. 5a and Supplementary Table 6). Similarly, in the individual learning condition, a comparable whole-brain analysis indicated that prediction errors were positively correlated with activation in the ventral medial prefrontal cortex, striatum, and parietal cortex (see Fig. 5b and Supplementary Table 7).

**Fig. 6:**
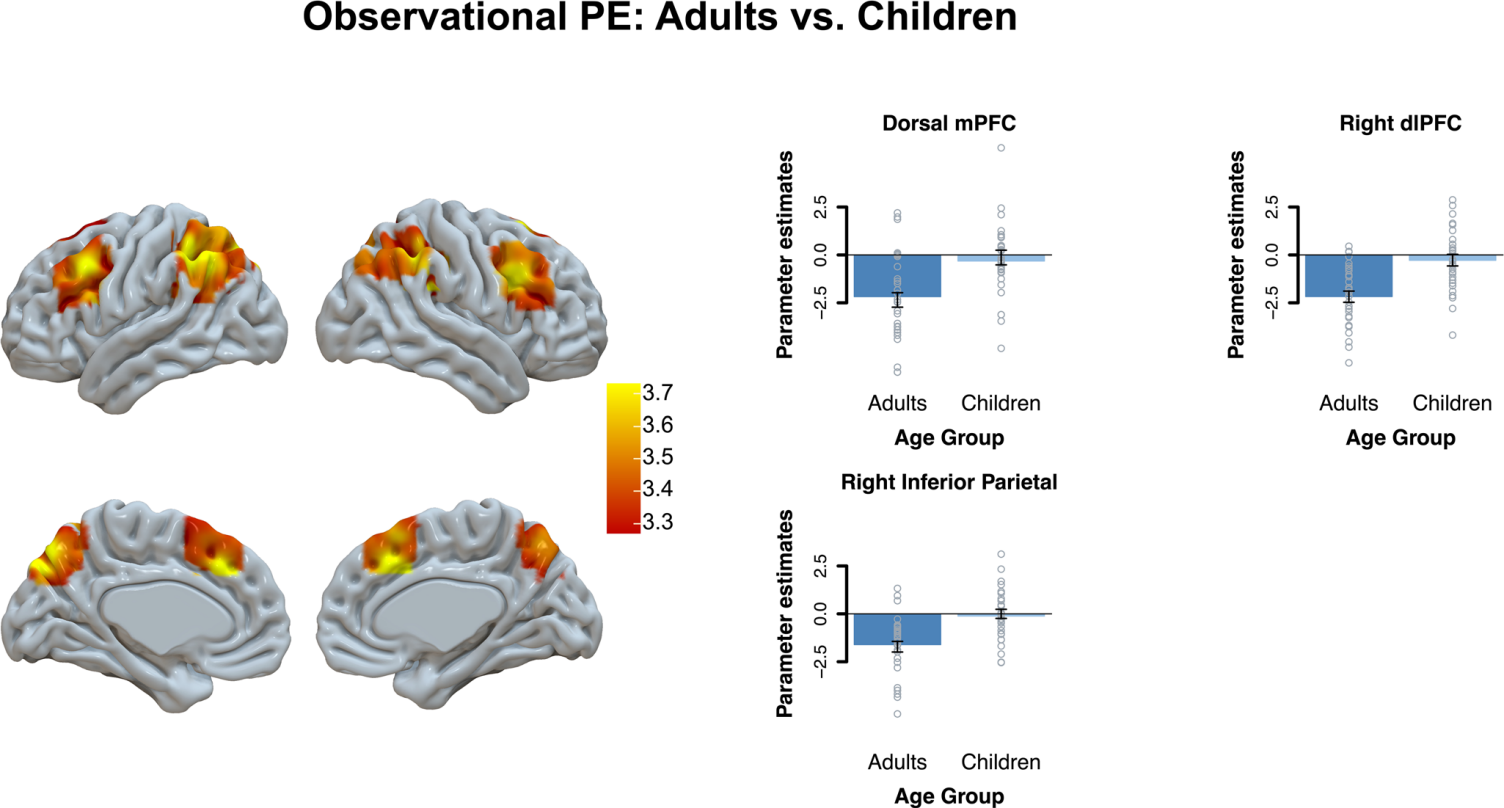
Age differences observational PEs: adults vs. children. This figure displays age-group differences in observational PEs with a whole-brain *t*-test at *qFDR* < 0.05 with a primary voxel-wise threshold of *p* < 0.001. For visualization we extracted the beta-values from the whole-brain effects per age-group. Error bars reflect the SEM.

Since our focus was on age differences, we then compared age groups in prediction error-associated brain activation for the observational and individual learning conditions. A whole-brain *t*-test comparing children versus adults revealed that observational prediction errors were more strongly represented in adults than in children in the dmPFC, dorsolateral PFC, right inferior parietal cortex, and right insula (see Fig. 6 and Supplementary Table 8). As can be seen in Fig. 6, more negative prediction errors resulting in stronger PE-related activation. We also compared age groups in the IL condition, but no significant age differences were observed.

#### Brain-behavioral relations: relating learning performance to prediction error activation

Lastly, we investigated the extent to which age differences in learning behavior were associated with prediction-error-related brain activation. To achieve this, we extracted parameter estimates from clusters responsive to both individual and observational learning conditions (as shown in Fig. 5), as well as clusters indicating an age-by-condition interaction (i.e., TPJ; as shown in Fig. 4). We employed a linear regression analysis for each region of interest to examine the relationship between prediction error activation and learning accuracy in both individual learning and observational learning conditions. Additionally, we included interactions with age groups to assess whether this relationship varied across different age groups (further details can be found in the Methods section and Supplementary Table 9). To account for multiple linear regressions, we applied an FDR correction across all behavior-brain regression analyses.

**Fig. 7:**
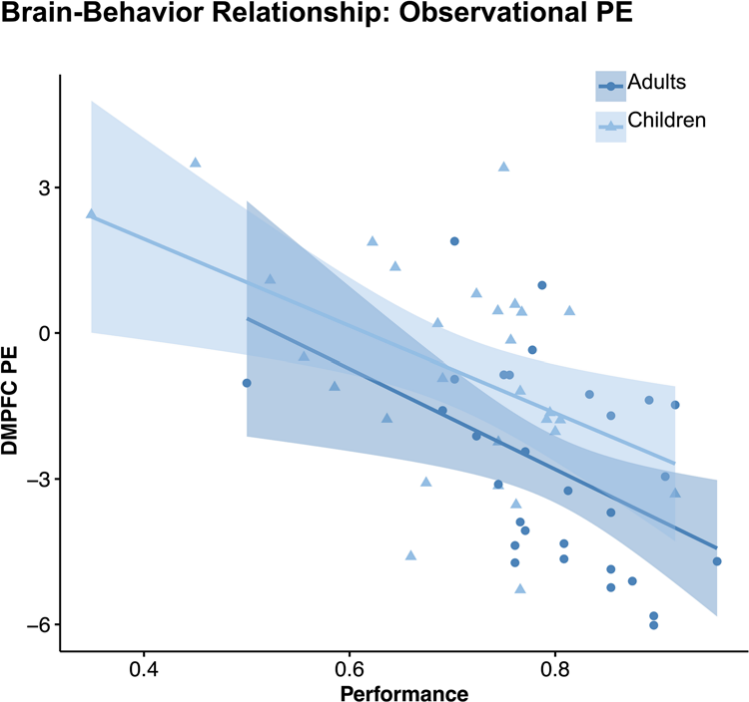
Brain-behavior relationship: observational PE. Scatter plot showing that more negative PE-related activation in the dmPFC was related to better performance during observational learning for both age groups. Shaded areas reflect 95% confidence intervals.

Learning accuracy in the observational learning condition was found to be correlated with observation prediction-error activation in the dmPFC (β = −1.12, t = −3.79, *p* < 0.001; see Fig. 7) and the right dlPFC (*p* = 0.037; see Supplementary Fig. 2a), but not with the right inferior parietal cortex or the right insula (*p*’s > 0.596; see Supplementary Table 9 for further details). Only the brain-behavior relationship in the dmPFC (not dlPFC) remained significant after multiple comparison correction. This association between dmPFC activation and task performance was consistent across age groups (performance × age group: *p* = 0.558). Thus, improved learning in the observational learning condition was linked to a stronger prediction-error response in the dmPFC (see Fig. 7) for both children and adults. It is important to note that individual learning performance was included as a covariate in this regression analysis, indicating that this association appears to be specific to observational learning performance (Supplementary Table 9).

Learning accuracy in the individual learning condition, after controlling for observational learning performance, was associated with individual prediction error activation in the left parietal cortex (β = 0.98, *p* = .007; see Supplementary Fig. 2b). However, this relationship did not survive multiple comparison correction. No significant relationships were observed between individual learning accuracy and activation in the vmPFC or striatal regions, nor in the right inferior parietal/TPJ (*p*’s > 0.076; Supplementary Table 9).

## Discussion

In this study we examined the behavioral and neural mechanisms underlying observational and individual reinforcement learning in 8–10-year-old children and young adults. Overall, we found that adults compared to children showed faster learning, better performance, and they were more optimally following the value of choice options across learning conditions (see Fig. 2). As expected, both age groups benefitted from observing other’s choices and outcomes. However, in contrast to our expectations, adults and children benefited to a similar degree from observing others behavior during learning and learning in observational versus individual conditions did not vary across development. To better understand the computational and neurobiological processes underlying learning we used reinforcement learning (RL) model in combination with fMRI. We observed that behavioral updating (as indicated by learning rates derived from the RL model fitting) did not differ across condition, and age, and both conditions and age groups showed higher learning rates from positive compared to negative feedback in both learning conditions. Choice behavior in children was generally less value-driven than in adults, and therefore showed more random, or potentially more exploratory, choice behavior. In addition, choice behavior was more value-driven in the observational than individual learning. Model-based parametric fMRI analyses complemented these behavioral insights. Observational and individual prediction errors were reflected in partly distinct (vmPFC, striatum), and (for adults) in partly overlapping brain regions, such as the temporal-parietal junction. Age-related differences were observed in observational prediction error coding in the dorsal medial prefrontal cortex, dorsal-lateral prefrontal cortex, parietal cortex, and insula cortex; regions closely related to cognitive control, social cognition, and social learning^24,30^. Moreover, only observational prediction error signals in the dmPFC correlated with observational compared to individual learning performance in both children and adults. This finding underlines the relevance of the dmPFC when learning from others.

Our behavioral results supported previously observed age-related differences in instrumental learning. In line with other developmental studies adults outperformed children in both individual and observational learning conditions. Children learned slower than adults^10,36–40^ and their choice behavior was more stochastic and less value-driven^41,42^. This decrease in stochasticity across age is one of the most consistent findings observed in reinforcement learning^7,8^. Potentially, this may relate to an increase in maintaining sustained attention, or improved working memory, accelerated by a developmental improvement in cognitive control. Alternatively, children may be more stochastic because they are more explorative than adults. Theoretical work suggested that higher exploration provides children with more learning opportunities and could allow them to quickly discover changes in environments^43^. With the current task design, stochasticity and exploration cannot be easily distinguished. Whether age-related differences in stochasticity are valuable for exploration in social- and non-social learning environments, should be addressed in future studies. Interestingly, our behavioral findings indicate that both children and adults can learn from observing other’s outcomes: All ages increase their updating after positive and negative feedback during observational learning, and all age groups benefit in performance in observational compared to individual learning. The adults’ advantage in reinforcement learning may therefore be reflected in more value-driven choice behavior.

Our brain-based results complement and extend these behavioral findings. First, our results showed age-related differences in the temporal-parietal region. Unexpectedly, this region was sensitive to observational and individual prediction errors in adults (although differently signed), but not in children. In adults, prediction errors correlated positively with brain activation in the TPJ during individual learning (indicating greater positive prediction errors resulted in greater activation), and negatively with brain activation during observational learning (indicating greater negative prediction errors resulted in greater activation). This supports findings supports prior findings that the TPJ is associated with other’s and one’s own associations^44^, self-other distinctions^45^ and that it can be linked in a valence-specific way to social and self-related prediction errors. However, in the current study prediction error related activation in the TPJ did not relate to age-related differences in behavioral performance. Future research will therefore need to replicate and extend these findings on the role of the TPJ in instrumental learning.

Second, our findings showed that observational prediction errors were related to neural activation in frontal regions including the dorsal medial PFC and dorsolateral PFC. and that that these relations were stronger for adults compared to children. Our finding that the medial prefrontal region is related to observational prediction error signaling in adults and children concurs with a broader framework, which links the ACC and dmPFC to a variety of social learning and decision-making skills^24,30^, such as outcome prediction error for confederate’s advice^31^, mentalizing^32,46^, and egocentric and allocentric outcomes of social decisions^24,47^. It has also been suggested that the role of different sub-regions of the mPFC may be more specific than previously recognized, and a more rostral part of the anterior cingulate cortex gyrus and dorsomedial prefrontal regions could be particularly specialized for observational prediction errors^5,23,24,28,48,49^. In addition, we observed that prediction-error activation in dmPFC related to performance in the observational learning condition in both children and adults (see Fig. 7). This may indicate an important role of the dmPFC for behavioral improvements in observational learning across age, although this does not necessarily relate to the observed age-related improvement in instrumental learning. Interestingly, prediction-error activation in the observational learning condition mostly increased when outcomes for others were worse than expected (see also^11^). This finding may be interpreted in multiple ways. Possibly, the current setup led to an experienced competitiveness between the observer and observed individual, although it was explicitly instructed that participants were neither in competition nor dependent on the behavior of the observed other (e.g.^11,50^,). Alternatively, seeing others lose may simply be a stronger learning signal. These directional effects were present across frontoparietal regions, indicating that these regions may be jointly involved in social comparison processes.

Finally, individual learning situations resulted in specific prediction-error related activation in the vmPFC, the left lateral PFC, the bilateral striatum, and bilateral parietal cortex (see Fig. 3)^10,35,51^. This supports previous findings and highlights there may be limited age-related differences per se in prediction error activation^27,40^. Whether adolescence is a period of heightened activation in reward-related learning environments as suggested by other studies^52–54^, is something that future research using samples with a wider, and more continuous, age range should disentangle.

Although our results confirmed several age-related findings from previous studies on individual and observational learning^9,10^, some of the results were unexpected. For instance, in contrast to previous findings on observational learning^10^, we did not find age-related performance differences between learning conditions. It seems viable that differences to results of previous studies arise because of the administered paradigm to make the task amenable for fMRI. For instance, in the previous EEG-studies the task was more complex in terms of learning conditions (i.e., another third condition was included) and more difficult as the timing was faster (i.e., no jitters were included). Moreover, when administering a social and non-social learning conditions an important question is to what extent findings are specific to social learning or reflective of more general learning processes^48^. In our experimental setup children and adults learn indirectly from others. To create a social learning setting, we informed participants that they could learn from another participant they met before the experiment, and they observe the other’s photo and name during the experiment. Varying the age of the other participant, in a previous study^9^, using the same social learning setting and task, we demonstrated that similarity in age (same-aged child vs. young adult) between the observed player and the observer influenced both behavioral and neural responses in 8-10 year olds. Building on these findings and because participants judged others as being similar to themselves (see Methods) we argue that it is likely that the participants perceived the observational learning conditions as social learning conditions rather than conditions in which they simply received only more information. Based on these considerations we would like to a few recommendations for future developmental studies on observational learning:

First, future studies should take the social context into account in which children learn “indirect” observational learning (e.g., observing others’ choices and outcomes in absence of the other player) in comparison to direct observational learning (e.g., “directly” observing others’ choices and outcomes in presence of the other player). Secondly, it is important to compare active individual learning to observational learning, where participants learn purely passively from observed information (i.e., without the ability to evaluate this information immediately after the observational phase on a trial-by-trial basis, as was done in this study). This allows for a more direct comparison of both learning processes based on a similar amount of information, which is not achievable in the current task design. Finally, we observed that our age groups differed in fluid intelligence. Children showed higher age-normed intelligence scores than adults, which may have influenced age-related differences in performance and neural activation. Although controlling for intelligence in our behavioral analyses did not change our main findings, future studies may address to what extent task difficulties and intelligence relate to age-related differences in observational learning. For instance, learning condition differences associated with prediction error activation might be related to greater task difficulty and higher cognitive load when learning from own outcomes. This could be linked to higher neural activation as task difficulty increases^55–57^.

To summarize, our findings show that learning from the outcomes of others, particularly when outcomes were worse than expected, was related to neural activation in the dmPFC, dlPFC and temporal-parietal activation which was more pronounced in adults than children. Here, only the dmPFC related to performance in observational learning for both children and adults. These findings confirm and extend the functional role of the medial PFC to a social observational learning context and specify its functional relevance for social learning for children and adults.

## Methods

### Participants

Thirty 18–20-year-old adults and 30 8–10-year-old children participated in the study. Data of one child was excluded due to inability to complete the task. For occasional occurring head motion (Framewise displacements >0.5) volumes with motion were flagged and were not included in regressors of interest but instead modeled by nuisance regressors (i.e., censored), number of censored volumes regressors varied between 2–12 (<10% of volumes). The final sample consisted of 30 adults (Age Mean (SD) = 19.45 (0.86); 16 female) and 29 children (Age Mean (SD) = 9.71 (0.89); 18 female). All participants were right-handed, had normal or corrected-to-normal vision, were screened for MRI contra-indications, and had no neurological or psychological disorders. Prior to the experiment we obtained informed written consent from the participants and both parents (in case of children). The study was approved by the Ethics Committee of the Leiden University Medical Center (LUMC). All anatomical MRI scans were reviewed and cleared by a radiologist from the radiology department of the LUMC. No anomalous findings were reported.

Subjects participated in one experimental session in which we assessed psychometric covariate measures not specific to the current study, and observational learning performance inside the MR scanner. Participants were recruited through local advertisements and received a compensation. Participants’ intelligence (IQ) was estimated with the subsets ‘similarities’ and ‘block design’ of the Wechsler Intelligence Scale for Children, third edition (WISC-III^58^). For both age groups, estimated IQs were in the normal to high range (Adults: Mean IQ (SD) = 106.67 (8.82), Range = 87.5–122.5; Children: Mean IQ (SD) = 112.76 (11.96), Range = 87.5–132.5). IQ did differ between age groups: Children showed higher IQ scores than adults (*F* (1, 57) = 4.952, *p* = 0.03, *η*_*p*_^*2*^ = 0.08). IQ is controlled for in all behavioral analyses.

### Experimental design

We used a probabilistic reward-based observational learning paradigm^9,11^ (see Fig. 1; controlled by PsychToolBox-3^59^). Participants were asked to choose one out of two abstract stimuli^60^. One stimulus was associated with a high probability of receiving reward (80% gains, 20% losses) and one associated with a low reward probability (20% gains, 80% losses). Before participants could choose, they observed an age- and gender-matched peer (who they met before starting the task) choosing between the same two abstract stimuli. Participants were told that the other player had already performed the task and that they could observe the recorded choices. In order to assess the credibility of our social manipulation we assessed participants perception of the other player at the end of the experiment. Almost all participants (93%) reported that they paid attention to the other player and on a 7-point Likert-scale (see Supplementary Fig. 3), that watching the other helped them for learning and that they judged the other as highly similar to themselves, reliable and believable (*p*’s < 0.001; one-sampled Wilcoxon tests for non-parametric data against 3.5). Age groups did not differ in their rating (unpaired Wilcoxon tests for non-parametric data; *p*’s > 0.06). However, unbeknownst to participants the observed choices were computer generated using a RL model (see Supplementary Fig. 4). The computer-controlled behavior of the model players was associated with the objective percentage of probabilistic positive or negative outcomes associated with each of the stimuli (see supplementary files^9,10^). The amount of observable information of the other player was manipulated across two learning conditions: *Observational learning* (observing both, the other player’s actions and outcomes; short OL) and *individual learning* (observing neither actions nor outcomes of the other player; short IL). In each condition, the trials followed the general structure of an observational phase that was followed by an action phase. That is, in the OL condition the participant would be first presented with a fixation cross for a variable amount of time per trial (not shown in Fig. 1). This jitter varied exponentially from 1 s to 8 s and was followed by a picture of the other person (i.e., precue of 1 s) and the presentation of a stimulus pair. By pressing a button with the ring finger of the right hand the other’s choices were revealed. Responses had to be given within a 2 s window and indicated by a white selection frame (i.e., 2 s – response time; see Fig. 1), which was followed by a 1 s outcome display representing the outcome of the other’s choice. Then, the action phase started with a fixation cross (i.e., jitter of 1–8 s) and then participants viewed his/her own picture (1 s), after which the same stimulus pair was presented. Participants could choose either the left or the right stimulus by pressing a button with the index or middle finger of the right hand. Again, responses had to be given within a 2 s window, which was followed by a 1 s outcome display. If no response was given within 2 s, in either the observational or the action phase of any condition the words “too slow” were presented on the screen. This happened rarely for adults (*M*trials = 2.41, *SD* = 2.27) and children (*M*trials = 6.89, *SD* = 5.54).

The IL condition followed the same timing and structure as the OL condition and participants also pressed a button when stimuli were presented in the observational phase, yet no choice (i.e., in this case both possible choices were surrounded by a white selection frame) or outcome information of the other were presented. Each condition was associated with two unique stimulus pairs per run and the order of conditions was mixed. In total, every pair was presented for eight trials per run (resulting in 32 trials and four abstract stimulus pairs per run). The four stimulus pairs (two per condition) per run were presented intermixed. Participants played in total three runs of approximately nine minutes each with 48 trials per learning condition (resulting in 96 trials with 16 abstract stimulus pairs in total). For five participants one run was excluded in further analyses because of high occasional motion (>10% of censored volumes per run). Participants were instructed to earn as many points as possible (as indicated by receiving a positive outcome signal) but were also informed that it was not possible to gain points on every trial, clarifying the probabilistic nature of the task. Before performing the task in the MRI, participants practiced the task for one run length.

### Reinforcement learning models

Learning action-outcome-contingencies can be computationally captured using RL models^18^. During RL learning the discrepancy between expected outcome on trial (t), Q_a_(t), and the actually received outcome on trial (t), r(t), is called prediction error (PE):

1 $${\rm{Prediction}}\,{\rm{error}}={\rm{r}}\left({\rm{t}}\right)-{{\rm{Q}}}_{{\rm{a}}}\left({\rm{t}}\right)$$

If outcomes are better (worse) than expected, the model will generate a positive (negative) PE, which is used to increase (decrease) the predicted value, *Q*_*a*_(*t*), associated with the chosen option *a* in the current trial *t*. Positive and negative outcomes received in each trial are used to update the predicted values of both options *a* (Eq. (2)) and *b* (Eq. (3))^61,62^:

2 $${Q}_{a}\left(t+1\right)={Q}_{a}\left(t\right)+\alpha [r\left(t\right)-{Q}_{a}\left(t\right)]$$

3 $${Q}_{b}\left(t+1\right)={Q}_{b}\left(t\right)+\alpha [-r\left(t\right)-{Q}_{b}\left(t\right)]$$

Thus, for the value of the unchosen option *b* (Eq. (3)) the counterfactural outcome is taken into account. The impact of the PE’s on forming new expectations is scaled by the learning rate *α*. A high learning rate (~1) indicates that a new experience (i.e., PE) has a stronger impact on future predictions whereas a low learning rate (~0) means that a PE only weakly influences the expected value and thereby choice behavior. Based on previous developmental studies, we included two independent learning rates for positive (α_pos_) and negative outcomes (α_neg_) to describe the learning behavior in the individual condition across development^40,63^.

For the OL condition, we extended the RL-algorithm used in the IL condition to describe social influences during learning: Learning from other’s choices and outcomes (see supplementary files for model comparisons and model recovery) was best captured by using a dual-update model, one updating phase for the observational (Eq. (4)) and one for action stage (Eq. (5)) as described in^64^.

4 $${Observational\; stage}\!:\,{Q}_{a}\left(t+1\right)={Q}_{a}\left(t\right)+\alpha [{r}_{{Other}}\left(t\right)-{Q}_{a}\left(t\right)]$$

5 $${Action\; stage}\!:\,{Q}_{a}\left(t+1\right)={Q}_{a}\left(t\right)+\alpha [{r}_{{Own}}\left(t\right)-{Q}_{a}\left(t\right)]$$

Similarly, to the RL models used in the individual condition we included two independent learning rates (α_pos_, α_neg_) in the OL condition. A higher learning rate thus means a quicker updating of expected values and thereby faster learning. For both stages and conditions, the probability of choosing option *a* from a stimulus pair (ab) was computed using a softmax function (Eq. (6))^34^:

6 $${P}_{a}=\frac{{e}^{{Q}_{a(t)}\times \beta }}{{e}^{{Q}_{a(t)}\times \beta }+{e}^{{Q}_{b(t)}\times \beta }}$$

The probability of selecting option *a* is influenced by the expected value *Q* of option *a* in trial *t* divided by the sum of the expected values of all possible options (*a and b*). The *β* parameter in this equation reflects the sensitivity of the subject to differences in expected value. Here, a lower *β* parameter indicates more stochastic responding.

For model comparisons we evaluated a set of alternative RL-algorithms (i.e., baseline RL-model with one learning rate (α), model with separate learning rates for positive (α_pos_) and negative outcomes and (α_neg_)^40,65^ and a model with separate learning rates for observational and action stage; see supplementary files, Supplementary Table 10 and Supplementary Fig. 5). All learning rates (α, α_pos_ and α_neg_) and the noise parameter (*β*) per condition were individually estimated by fitting the model predictions to participants’ choices (see supplementary files for further details on the model fitting procedure). The *β* parameters were fit with constraints between [0 5]. The α parameters were constrained between [0 1]. For model selection purposes, we computed the Bayesian information criterion (BIC) across all subjects for the different models, where lower BIC values indicate better fit (see ref. 66). For both learning conditions (i.e., IL and OL) the best fitting model across all subjects included two independent learning rates (α_pos_, α_neg_) and the noise parameter (*β*) (see supplementary files for further details and S2 for an overview of all model comparisons and parameter estimates per model). For the model-based fMRI analyses we used the median parameter estimates per age group (Fig. 2) of the best fitting model per learning condition to calculate trial-by-trial PEs^11,62,67–69^. In imaging analyses PEs were scaled and mean-centered.

To explore the validity of the RL models and the model selection procedure, we performed 1) model and 2) parameter recovery analyses (see supplementary files and Supplementary Fig. 6 for further details). As part of quality control, we further performed simulations using the individual parameter estimates for each subject for the best fitting model. The simulations indicated that the best fitting model per condition was able to capture learning on a trial-by-trial level in each age group (see supplementary files for further details and Supplementary Fig. 7).

### Behavioral data analysis

Choice behavior was analyzed using a mixed-effects generalized linear model as implemented in the lme4 package in R^70^. Accuracy (proportion of optimal choice) was averaged across runs and modeled using the between-subjects predictor age group and the within-subjects predictors learning condition (IL, OL) and trial number (1:8).

Mixed effects model formula:

7 $$\begin{array}{l}{Accuracy} \sim {age\; group}* {learning\; condition}* {trial}+{intelligence}\\\qquad\qquad+(1+{learning\; condition}+{trial}{\rm{|}}{id})\end{array}$$

We treated the between-subjects predictor age group as a fixed effects factor, whereas all within-subjects’ predictors of interest were treated as fixed and random effects at the individual subject level. We additionally included intelligence as predictor to control for age differences in intelligence. The categorical predictors were contrast-coded, the continuous predictor trial was mean-centered. Regression weights (beta values), z-values and corresponding p-values are reported (see Supplementary Table 1).

The learning rates and inverse temperature of the best fitting models per conditions across age were significantly non-normally distributed (*p*’s < 0.05). Thus, we used robust mixed effects model to test for age and condition effects on learning rates and inverse temperature, respectively as implemented in the robustlmm package (see Supplementary Tables 2 and 3). Note that due to convergence problems we use a simpler fixed and random effect structure in these models (see Eq. (8)).

Mixed effects model formula (example):

8 $${Learning\; Rate} \sim {age\; group}* {condition}+{age}\,{group}* {valence}+{condition}* {valence}+{intelligence}+(1{\rm{|}}{id})$$

Finally, we assessed whether behavior per condition (i.e., accuracy) related to condition-specific prediction-error activation. We used multiple linear regression models predicting PE activation by proportion of optimal choice (accuracy), age, and their interaction, controlling for learning in the other condition, and intelligence. The categorical predictors were contrast-coded, the continuous predictor trial was mean-centered. Regression weights (beta values), z-values and corresponding p-values are reported (see supplementary files and Supplementary Table 9). *P*-values in brain-behavioral analyses were considered significant when evaluated against an FDR-corrected threshold that included all effects across the multiple ROIs (8) that were examined.

Multiple linear models’ formula (example):

9 $${PE}\,{activation}\,{OL} \sim {age}\,{group}* {accuracy}\,{OL}+{accuracy}\,{IL}+{intelligence}$$

### MRI data acquisition

MRI data were acquired with a standard whole-head coil using a 3-T Philips Achieva scanner. T2*-weighted echoplanar images (EPIs) were obtained during three functional runs, in which the first two volumes were discarded to allow for equilibration of T1 saturation effects. Volumes covered the whole brain (38 slices; 2.75 mm slice thickness; ascending acquisition) and were acquired every 2200 ms (TE = 30 ms). A high resolution T1-weighted anatomical scan was included at the end of the imaging protocol (140 slices; TR = 9.76 ms; TE = 4.59 ms; flip angle = 8°; FOV = 224 × 177.33 × 168 mm; in-plane resolution = 0.875 × 0.875 mm; slice thickness = 2 mm). Visual stimuli were projected onto a screen that was visible for participants via a mirror attached to the head coil. Before the experiment, children were trained with a mock-scanning procedure. All participants were reminded during the session not to move during scanning, and head motion was restricted by using foam padding.

### fMRI preprocessing and model specification

Data preprocessing and analysis were conducted using SPM8 (Welcome Department of Cognitive Neurology, London). Images were corrected for differences in timing of slice acquisition, followed by rigid body motion correction. The T1 structural image was co-registered to the functional images and segmented according to gray matter, white matter, and cerebrospinal fluid. Functional images were then spatially normalized using the normalization parameters obtained from the segmentation procedure. The normalization algorithm used a 12-parameter affine transformation together with a nonlinear transformation involving cosine basis functions. During normalization the data was re-sampled to 3-mm cubic voxels. Templates were based on the MNI305 stereotaxic space^71^. Functional volumes were smoothed with a 6-mm full-width at half maximum isotropic Gaussian kernel. Statistical analyses were performed on individual subjects’ data using the General Linear Model (GLM).

To investigate the neural responses to own and other’s outcomes and PE’s, we modeled in separate regressors the onset of the choice stimuli with the reaction time as the duration in the observational and the action phase for both conditions. Choice value, derived from the reinforcement learning model, was included as a parametric modulator of the choice regressor in the observational (OL), and of the choice regressor in the action phase (OL, IL). The onset of the outcome was modeled with a stick function. Separate outcome regressors were created for own and other’s outcomes in the OL condition, and for own and no-outcomes in the IL condition. In addition, three outcome regressors (own and other’s outcomes in the OL, and own outcomes in the IL condition) included a parametric modulation of trial-wise PE’s derived from the reinforcement learning model. Trials in which participants did not respond on time and censored motion trials were modeled separately as regressors of no interest. Finally, 6 head-motion parameters were included as nuisance regressors.

Our main analyses include the comparison between own outcomes in the IL (action phase), and other’s outcomes in the OL condition (observational phase, see Fig. 1). For completeness we include whole-brain maps of the non-modulated feedback event in Supplementary Fig. 8. As a control analysis to investigate whether substantial differences would arise when considering one’s own outcomes in the IL and OL conditions, we compared PE-related activation for self-outcomes in both conditions (further details can be found in the supplementary files and Supplementary Fig. 9). It is important to note that PE activation patterns largely overlapped, and no significant differences were observed. We chose to use data from the IL condition exclusively to maintain a similar number of trials entered into the analysis. Also, the action phase in the IL condition is less affected by the information from the observational phase.

Unless stated otherwise, whole-brain results comparing learning conditions were considered significant if they exceeded an FWE cluster-corrected threshold of *p* < 0.05, with an initial threshold of *p* < 0.001. Age-related differences were tested with an FDR cluster-corrected threshold of *p* < 0.05, with an initial threshold of *p* < 0.001. We used the MarsBaR toolbox^72^ for SPM8 to extract beta-values from cluster of activation observed in our contrasts of interest, which were used in subsequent correlations with performance and parameter estimates.

### Reporting summary

Further information on research design is available in the Nature Research Reporting Summary linked to this article.

### Supplementary information

Supplementary Files

Reporting Summary

## Data Availability

The data that support the findings of this study can be found in the Leiden Repository (10.34894/W4WMPZ).

## Code availability

All relevant R codes can be found in the Leiden Repository (10.34894/W4WMPZ).
