# Supplementary material for: Observational reinforcement learning in children and young adults
Source: NPJ Sci Learn. 2024 Mar 13;9:18. doi: 10.1038/s41539-024-00227-9 (PMC10937639; doi:10.1038/s41539-024-00227-9)
Supplement: Supplementary file 1 — Supplementary Files [file 41539_2024_227_MOESM1_ESM.pdf]

Supplementary Files for

Observational reinforcement learning in children and adults

Julia Rodriguez-Buritica[1,2,5], Ben Eppinger[1,3,4,5], Hauke R. Heekeren[1,6], Eveline A. Crone[7,8,9], & Anna C.K. van Duijvenvoorde[8,9]

[1]Department of Education and Psychology, Freie Universität Berlin, Berlin, Germany; [2]Berlin School of Mind and Brain & Department of Psychology, Humboldt University of Berlin, Germany; [3]Department of Psychology, Concordia University, Montreal, Canada; [4]Department of Psychology, Technische Universität Dresden, Dresden, Germany; [5]Department of Psychology, University of Greifswald, Greifswald, Germany; [6] Executive University Board, Universität Hamburg, Hamburg, Germany; [7]Department of Psychology, Education and Child Studies, Erasmus University Rotterdam, Rotterdam, Netherlands; [8]Institute of Psychology, Leiden University, Leiden, The Netherlands; [9]Leiden Institute for Brain and Cognition, Leiden, The Netherlands

This PDF file includes:

Supplementary text
Supplementary Tables 1 to 10
Supplementary Figures 1 to 9
Supplementary References for Supplementary reference citations

Supplementary Results

(1) Supplementary Tables

Summaries of linear mixed effects regression

Supplementary Table 1. **Condition and age-related differences in optimal choice (accuracy) controlling for IQ.** Summary of linear mixed effects regression.

	B(SE)	*SE*	*P*
Intercept	0.58	0.02	< .001
Age	0.10	0.03	< .001
Condition	0.12	0.02	< .001
Trial	0.04	0.01	= .005
Age * Condition	-0.001	0.03	= .956
Age * Trial	0.04	0.02	= .031
Condition * Trial	-0.04	0.02	= .026
Age * Condition * Trial	-0.03	0.03	= .267
IQ-Total	0.01	0.01	= .699

$R^2 = .31$

Supplementary Table 2. **Condition and age-related differences in learning rate controlling for IQ.**

Summary of robust linear mixed effects regression.

	B(SE)	*SE*	*P*
Intercept	0.01	0.22	= .981
Age	0.01	0.31	= .978
Condition	0.12	0.22	= .606
Valence	0.75	0.22	< .001
Age * Condition	0.12	0.32	= .698
Age * Valence	0.09	0.32	= .779
Condition * Valence	-0.32	0.28	= .253
IQ-Total	0.02	0.01	= .219

Supplementary Table 3. **Condition and age-related differences in inverse temperature controlling for IQ.**

Summary of robust linear mixed effects regression.

	B(SE)	***SE***	***P***
Intercept	0.69	0.14	< .001
Age	0.91	0.20	< .001
Condition	0.95	0.13	< .001
Age * Condition	0.009	0.18	= .958
IQ-Total	0.08	0.05	= .134

Supplementary Table 4. **Significant clusters of activation of individual > observational prediction-errors.**

Cluster location	MNI X (mm)	MNI Y (mm)	MNI Z (mm)	*k*	Peak *z* score
Right Caudate	15	17	4	878	5.55
Right Caudate	9	17	-8		5.18
Left Caudate	-15	20	-5		4.86
Right Angular Gyrus	60	-55	28	120	5.10
Right Inferior Parietal Lobule	60	-52	43		4.73
Right Angular Gyrus	57	-61	34		3.94
Left Inferior Parietal Lobule	-51	-55	46	338	4.43
Left Inferior Parietal Lobule	-54	-40	55		4.11
Left Inferior Parietal Lobule	-57	-34	49		4.07
Left Frontal Inferior Triangularis	-48	41	7	70	4.21
Left Frontal Inferior Triangularis	-45	35	28		4.06
Left Frontal Inferior Triangularis	-45	32	16		3.82
Left Medial Superior Frontal Gyrus	-9	65	4	75	4.19
Left Anterior Cingulum	-3	50	1		3.63
Left Medial Superior Frontal Gyrus	-9	68	13		3.45

Montreal Neurological Institute (MNI) coordinates denote the peak and subpeaks of each cluster (Whole-brain FWE cluster-level corrected (pFWE < .05), with a primary voxel-wise threshold of p < .001).

Supplementary Table 5. **Significant clusters of activation in relation to the interaction between condition (individual > observational) and age (adults > children).**

Cluster location	MNI X (mm)	MNI Y (mm)	MNI Z (mm)	*k*	Peak *z* score
Left Inferior Parietal Lobule	-48	-40	37	67	3.99
Left Supramarginal Gyrus	-54	-49	28		3.50
Right Middle Occipital Gyrus	27	-73	31	78	3.95

Montreal Neurological Institute (MNI) coordinates denote the peak and subpeaks of each cluster (Whole-brain FWE cluster-level corrected (pFWE < .05), with a primary voxel-wise threshold of p < .001).

Supplementary Table 6. **Significant clusters of activation in relation to observational prediction-errors in the observational learning condition.**

Cluster location	MNI X (mm)	MNI Y (mm)	MNI Z (mm)	*k*	Peak *z* score
Right Angular Gyrus	57	-55	37	15	5.35
Right Angular Gyrus	57	-55	28		4.89
Right Frontal Inferior Operculum	39	17	34	34	5.35
Right Middle Frontal Gyrus	45	20	40		5.06
Right Frontal Inferior Operculum	45	11	40		4.99
Right Supplementary Motor Area	3	20	46	8	4.93
Right Insula Lobe	33	20	-2	7	5.00

Montreal Neurological Institute (MNI) coordinates denote the peak and subpeaks of each cluster (Whole-brain F-test, FWE voxel-level corrected, pFWE < .05, k>3).

Supplementary Table 7. **Significant clusters of activation of individual prediction-errors in the individual learning condition.**

Cluster location	MNI X (mm)	MNI Y (mm)	MNI Z (mm)	*k*	Peak *z* score
Left Medial Superior Frontal Gyrus	-6	65	1	141	5.68
Right Orbital Medial Frontal Gyrus	0	53	-2		5.46
Left Medial Superior Frontal Gyrus	-15	65	4		5.42
Left Middle Occipital Gyrus	-45	-70	25	31	5.32
Left Middle Occipital Gyrus	-39	-76	28		4.94
Left Middle Occipital Gyrus	-33	-70	31		4.85
Left Olfactory Bulb	-15	8	-14	8	5.21
Left Inferior Temporal Gyrus	-57	-52	-11	6	5.16
Right Caudate	9	14	-8	5	4.84

Montreal Neurological Institute (MNI) coordinates denote the peak and subpeaks of each cluster (Whole-brain F-test, FWE voxel-level corrected, *p*FWE < .05, *k*

Supplementary Table 8. **Significant age differences in clusters of activation in relation to observational prediction-errors (adults > children)**

Cluster location	MNI X (mm)	MNI Y (mm)	MNI Z (mm)	*k*	Peak *z* score
Left Precuneus	-9	-76	43	104	4.56
Right Precuneus	3	-57	55		
Left Inferior Parietal Lobe	-48	-49	49	291	4.46
Left Inferior Parietal Lobe	-42	-52	37		
Right Inferior Parietal Lobe	42	-40	37	143	4.24
Right Inferior Parietal Lobe	45	-46	43		
Right Inferior Frontal Gyrus	42	14	34	142	4.23
Right Middle Frontal Gyrus	54	17	34		
Right Middle Frontal Gyrus	42	23	40		
Left Middle Frontal Gyrus	-42	17	43	118	4.05
Left Middle Frontal Gyrus	-42	26	37		
Left Superior Medial Gyrus*	0	26	43	53	3.87
Left Supplemental Motor Area	0	23	55		

Montreal Neurological Institute (MNI) coordinates denote the peak and subpeaks of each cluster. Results displayed at FDR cluster-level correction with *qFDR* < .05 with a primary voxel-wise threshold of $p < .001$, k>3. All clusters, except the dmPFC (*) also survived a more stringent whole-brain FWE cluster-level corrected threshold (*p*FWE < .05), with a primary voxel-wise threshold of $p < .001$.).

Supplementary Table 9. **Brain-behavioral associations controlling for learning condition and IQ.** Summary of multiple linear regressions between PE activation and behavioral measures including accuracy (performance), age, and the interaction between accuracy x age.

	B(SE)	***SE***	***P***
**Observational PE in dmPFC controlled for IL**			
Intercept	-1.70	0.29	< .001
Performance	-1.50	0.49	= .003
Performance IL	0.47	0.47	= .325
Age	-0.59	0.30	= .054

Performance x Age	-0.18	0.31	= .558
IQ-Total	-0.14	0.29	= .621
R^2 = .38			
**Observational PE in dlPFC controlled for IL**			
Intercept	-1.49	0.24	< .001
Performance	-0.45	0.41	=.276
Performance IL	-0.09	0.40	= .817
Age	-0.64	0.25	= .015
Performance x Age	-0.09	0.27	= .731
IQ-Total	-0.20	0.24	= .429
R^2 = .28			
**Observational PE in parietal cortex controlled for IL**			
Intercept	-1.66	0.37	< .001
Performance	-0.16	0.34	= .644
Performance IL	-0.11	0.27	= .675
Age	0.15	0.55	= .793
Performance x Age	0.49	0.54	= .366
IQ-Total	0.30	0.25	= .236
R^2 = .04			
**Observational PE in right Insula controlled for IL**			
Intercept	-1.42	0.45	= .003
Performance	-0.22	0.41	= .596
Performance IL	0.56	0.33	= .091
Age	-0.40	0.66	= .548
Performance x Age	0.16	0.64	= .811
IQ-Total	0.49	0.30	= .107
R^2 = .1			
**Individual PE in inferior parietal cortex controlled for OL**			
Intercept	1.60	0.24	< .001
Performance	0.98	0.35	= .007
Performance OL	-0.56	0.38	= .144
Age	-0.23	0.28	= .407
Performance x Age	-0.04	0.26	= .868
IQ-Total	0.23	0.24	= .341
R^2 = .15			

**Individual PE in vmPFC controlled for OL**			
Intercept	1.55	0.46	< .001
Performance	0.21	0.45	= .643
Performance OL	-0.71	0.33	= .037
Age	1.15	0.67	= .091
Performance x Age	-0.25	0.62	= .693
IQ-Total	0.05	0.30	= .866

R^2 = .1

**Individual PE in right ventral striatum controlled for OL**			
Intercept	0.86	0.39	= .035
Performance	0.14	0.39	= .712
Performance OL	-0.28	0.29	= .339
Age	1.0	0.58	= .089
Performance x Age	-0.18	0.53	= .739
IQ-Total	0.30	0.25	= .249

R^2 = .07

**Individual PE the left ventral striatum controlled for OL**			
Intercept	1.31	0.37	< .001
Performance	0.32	0.37	= .395
Performance OL	-0.21	0.27	= .434
Age	0.53	0.54	= .338
Performance x Age	-0.63	0.51	= .215
IQ-Total	0.18	0.24	= .467

R^2 = .05

**Individual PE in TPJ/Inferior parietal cortex controlled for OL**			
Intercept	2.08	5.51	< .001
Performance	0.68	1.82	= .075
Performance OL	0.18	0.66	= .511
Age	-0.87	-1.59	= .117
Performance x age	-0.25	-0.49	= .630
IQ-Total	0.15	0.63	= .533

R^2 = .31

Abbreviations: Predication error (PE), individual learning (IL), observational learning (OL)

(2) Supplementary Figures

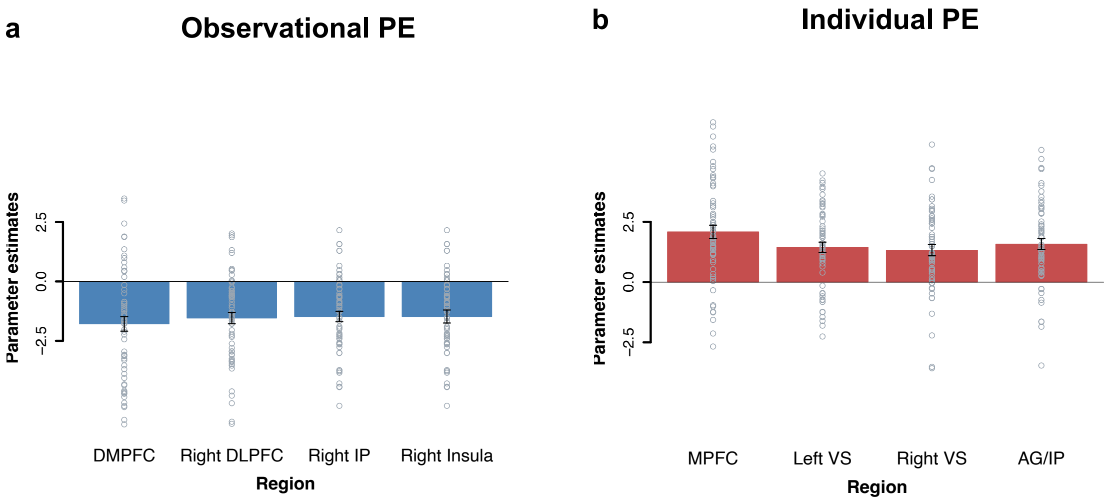

Supplementary Figure 1. **Activation clusters for Pes relative to (implicit) baseline** for **a** observational PE activation and **b** individual PE activation. For visualization purposes we extracted the beta-values from the whole-brain effects per learning condition. Abbreviations: DMPFC = dorsal medial prefrontal cortex, Right DLPFC = Right dorsal-lateral prefrontal cortex, Right IP = Right Inferior Parietal, MPFC = medial prefrontal cortex, Left/Right VS = Left/Right Ventral Striatum, AG/IP = Angular Gyrus/Inferior Parietal. Error bars reflect the SEM.

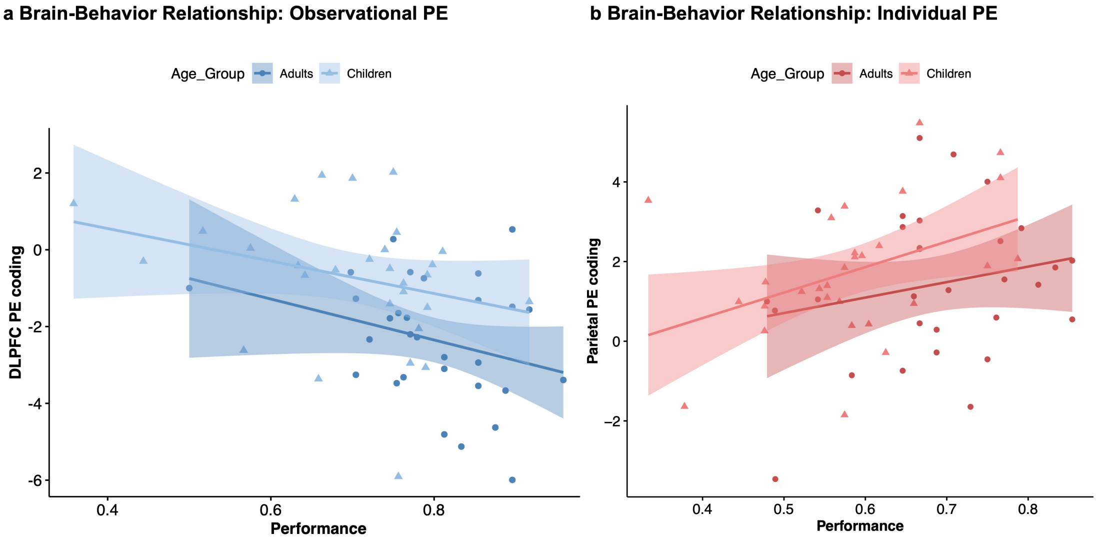

Supplementary Figure 2. **Brain-behavioral relations**. Scatter plot showing that **a** more negative PE activation in the dlPFC was related to better performance during observational learning for both age groups and **b** more positive PE activation in the parietal cortex was related to better performance during individual learning. Both did not survive multiple comparison correction (FDR). Shaded areas reflect 95% confidence intervals.

Supplementary Methods:

(1) Experimental Design

Model-generated choices of "model players". The choices of the "model players" presented to the participants were generated using a Q learning algorithm (see equation (1))[1,2]. Specifically, after a reward *r*, the value *Q* of action *a* in the next trial was calculated according to the delta updating rule:

$$Q_a(t+1) = Q_a(t) + \alpha\,[r(t) - Q_a(t)]$$

(1)

where α is the learning rate, *r(t)* the reward obtained after performing action *a* and *t* indexes the current trial. The probability of performing action *a* was computed using a softmax function (see equation (2)) [3]:

$$P(a) = \frac{e^{Q_a(t)/\beta}}{\sum_{c \in A} e^{Q_c(t)/\beta}}$$

(2)

where *P*(*a*) is the probability of choosing action *a*, *A* is the set of all possible actions and β is the temperature parameter that controls the competition between possible choices. The computer-controlled behavior of the model player (i.e., in the observational learning condition) was associated with the same percentage of probabilistic positive or negative outcomes (80% gains for the good, 20% for the bad choice) as experienced during the individual learning condition. To ensure comparability between age groups (see Supplementary Figure 2), the means of the rewards obtained by the model were constrained to small deviations from each condition's true mean with a 2.5% maximum deviation (between 77.5% and 82.5% upon choosing the good option and between 17.5% and 22.5% upon choosing the bad option).

The Q-values were set to zero at the beginning of the task and continuously updated on subsequent trials. The β and α parameters were estimated based on data from 30 subjects (acquired in a prior pilot test) from 10^5-simulated runs with the same model.

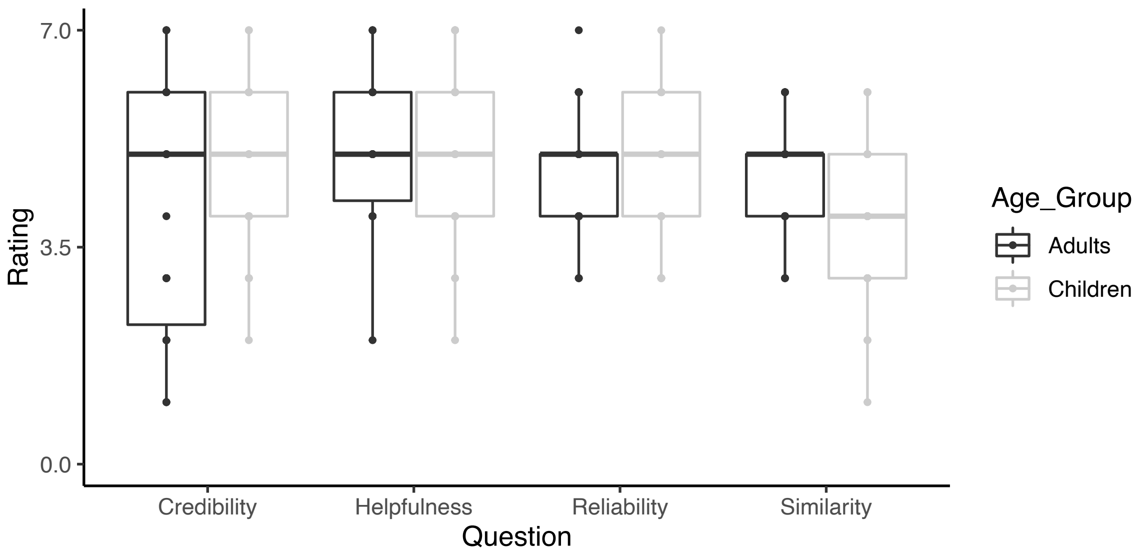

Supplementary Figure 3. **Results of exit questionnaire** for ratings of the other player per age group: Credibility: How much you believed the other? Helpfulness: How helpful was it to watch the other player? Reliability: How reliable was the other player? Similarity: How similar is the other to yourself?

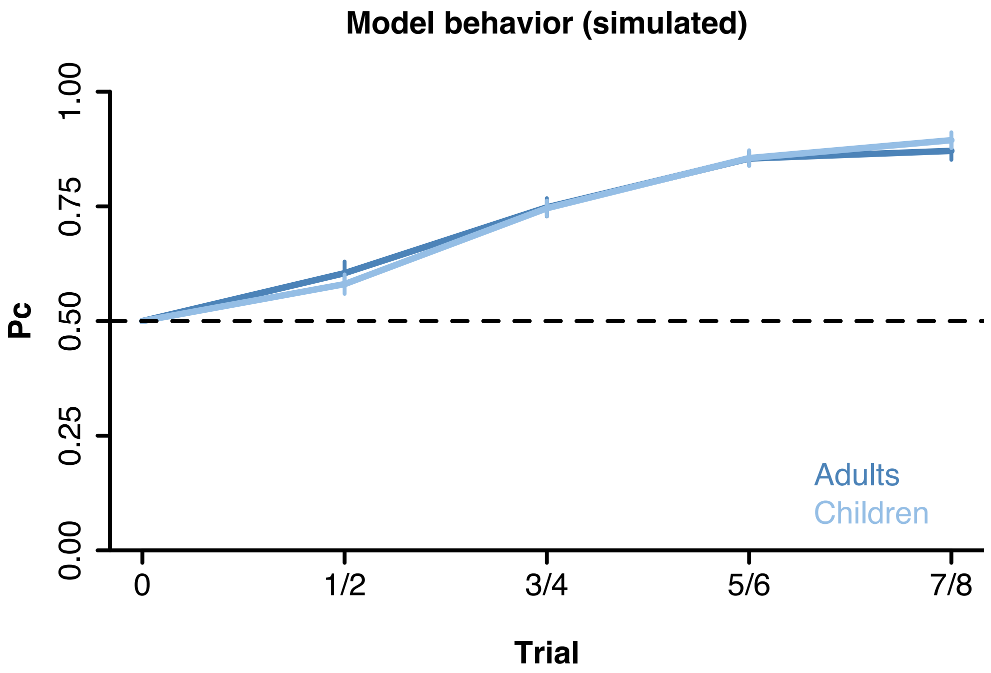

Supplementary Figure 4. **Computer-simulated averaged learning curve** for the observational learning condition per age group. Error bars reflect the SEM.

(2) Reinforcement Learning Models

Model Fitting Procedure and model comparison. All computational models were fit in R[4]. We used a L-BFGS-B algorithm implemented in the optim function in R to estimate the model parameters for each participant. We set all starting values for each of the parameters per age group to .5. For model comparisons we evaluated a set of alternative models including extensions to the basic RL-algorithm, described in the main text (see Supplementary Table 7 for an overview). This was done separately for the two learning conditions. For the observational learning condition, we fitted three additional RL-models: (I) a model with one learning rate (α; OL Base); (II) a dual learning rate (α_{pos} & α_{neg}; OL PN); and (III) a model with two independent learning rates (OL OA) for the observation (α_{other}) and for the action stage (α_{own}). For the individual learning condition, we compared (I) a model with one learning rate (α; IL Base) and (II) a dual learning rate (α_{pos} & α_{neg}; IL PN).

Supplementary Table 10. Overview of included model parameters for fitted models per condition

Condition	Model Name	PM	Number Estimated PM
Observation	Dual-update RL (OL Base)	α<br>β	2
Observation	Dual-update RL (OL PN)	α_{pos}<br>α_{neg}<br>β	3*
Observation	Dual-update RL (OL OA)	α_{other}<br>α_{own}<br>β	3
Individual	Single-update RL (IL Base)	α<br>β	2
Individual	Single-update RL (IL PN)	α_{pos}<br>α_{neg}<br>β	3*

* indicates best-fitting model

For model selection purposes, we computed the Bayesian Information Criterion (BIC), where lower BIC values indicate better model fit. Average BIC fits were compared for

across all participants: For both learning conditions the best model fit converged on the model with two independent learning rates for the positive outcomes (α_{pos}) and for the negative outcomes (α_{neg}) (with a lower BIC for the IL condition of 192 and for the OL condition of 313 as compared to the second-best fitting model; see Supplementary Figure 3). When comparing BICs within each age groups, the IL Base model fitted the children's data slightly better than the IL PN model (BIC difference of 14; see Supplementary Figure 3). That was not true in the observational learning condition, here the OL PN fitted the data across and within each age group the best. For combination with imaging analyses, we chose the best model across all participants (IL PN/OL PN).

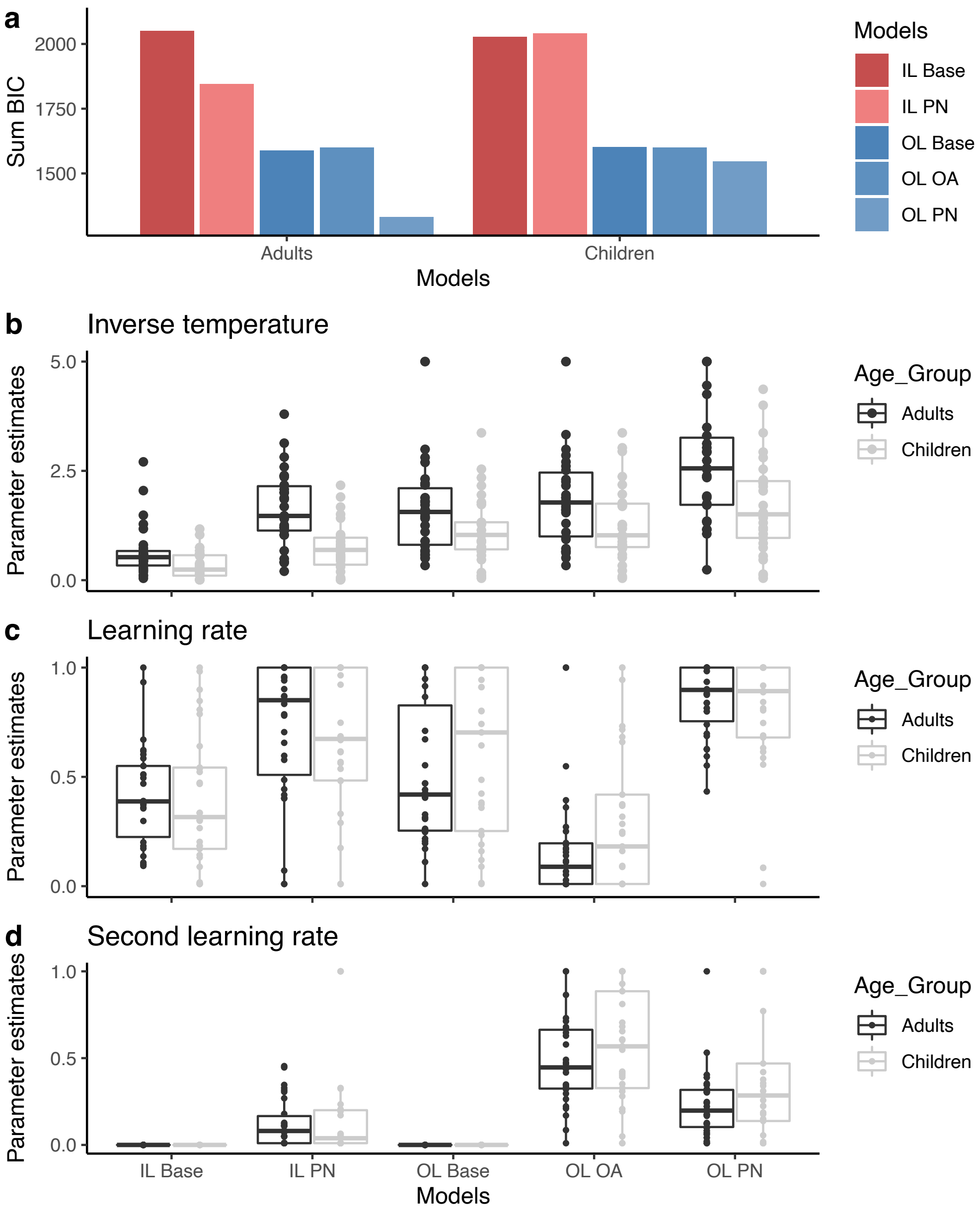

Supplementary Figure 5. **Model comparisons and parameter estimates. a** Cumulated Bayesian information criterion (BIC) of the computational models separately for age group and condition (individual learning (IL) and observational learning (OL)). Base = Baseline RL-model; PN = Model with separate learning rates for positive and negative outcomes; OA = Model with separate learning rates for observational and action stage. Parameter estimates per age group and computational model for the two conditions **b** inverse temperature (i.e., beta). **c** Learning rate (i.e., alpha) (for the PN models = positive learning rate; for the OA model = learning rate in action stage) **d** Second learning rate (for the PN models = negative learning rate; for the OA model = learning rate in observational stage).

Model and parameter recovery. To explore the validity of the RL models and the model selection procedure, we performed (1) model and (2) parameter recovery analyses. (1) For each of the 6 RL models we simulated 64 subjects based on 4 true values for each parameter. All RL models were successfully recovered (see Supplementary Figure 5 a). (2) Next, the parameters of the simulated dataset were estimated using the same fitting procedure as used for the subject data and compared to the true values. As can be seen in Supplementary Figure 5 b-d estimated parameter values were successfully estimated for each of the RL models. To compare whether the difference between true values and estimated parameter values did differ between RL models, we used a non-parametric Kruskal-Wallis ANOVA. The estimation biases (differences between estimated parameter values and true values) for the three parameters (two learning rates and decisions noise) were comparable across the different RL models (H's (3) > 0.1, p's > 1).

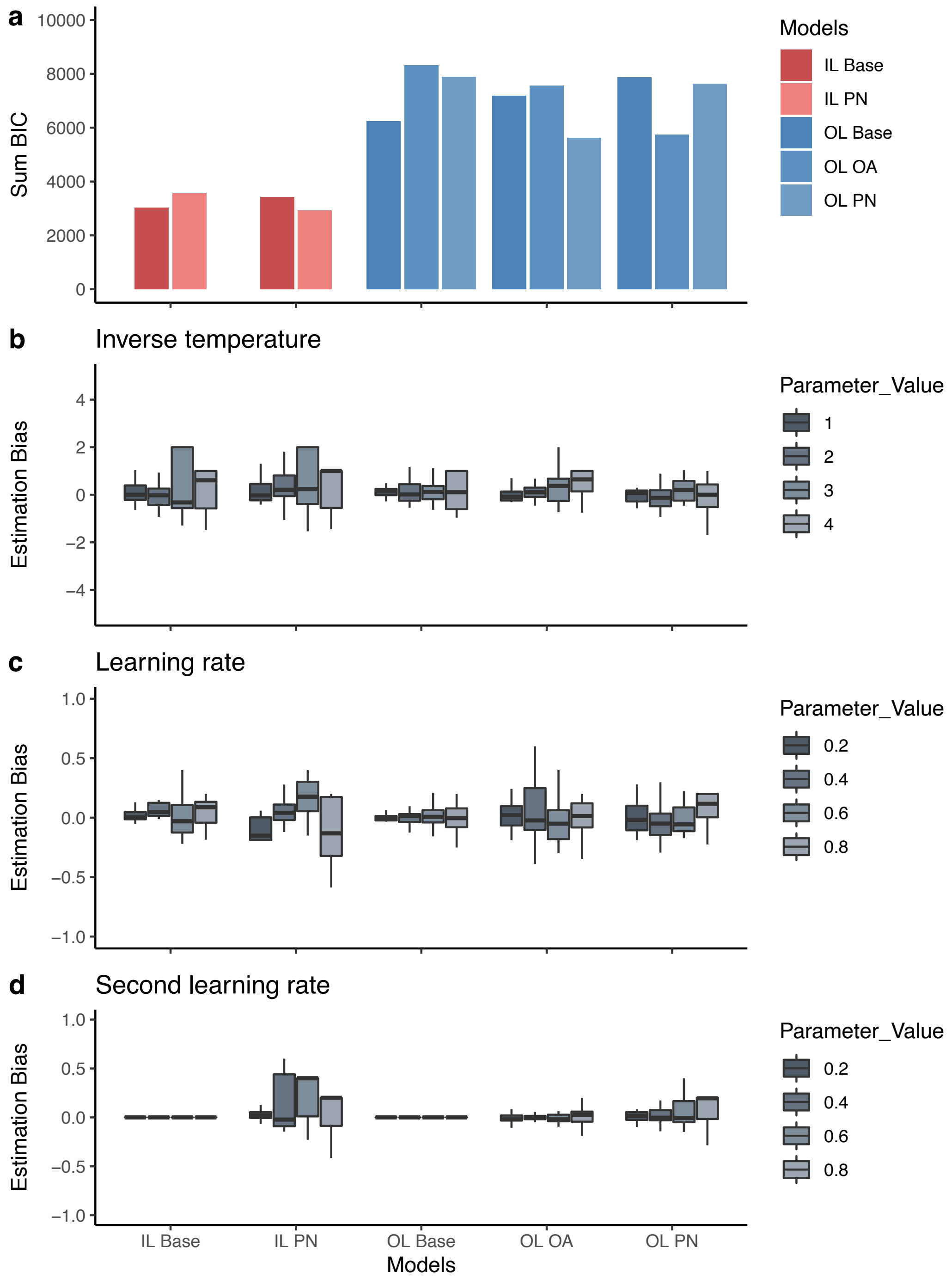

Supplementary Figure 6. **Model and parameter recovery. a** For a model-recovery analysis, data for each computational model was simulated using a broad range of model parameters. The simulated was evaluated using each computational model within each learning condition (i.e., individual learning (IL) and observational learning (OL)). Each model used to generate the data had lower Bayesian information criterion (BIC) than the other models that did not generate the data. This indicates that all models can reliably be recovered. Base = Baseline RL-model; PN = Model with separate learning rates for positive and negative outcomes; OA = Model with separate learning

rates for observational and action stage. **b** In a parameter-recovery analysis the simulated data was estimated using the same estimation pipeline as for the empirical data and computed the difference between estimate and generative parameters (estimation bias). The subplot shows the mean ± SD estimation bias for the beta parameter that modeled the inverse temperature. **c** Mean ± SD estimation bias for the alpha parameter that modeled the learning rate. **d** Mean ± SD estimation bias for the alpha 2 parameter that modeled the second learning rate for model 2 and 4 (i.e., negative learning rate) and within model 5 (i.e., separate learning rate for observational stage).

Posterior predictions of the best fitting models. As part of quality control, we further performed simulations using the individual parameter estimates for each subject for the best fitting model per condition, and this was done separately for the two age groups. For each condition, we simulated 30 agents for the adult and 29 agents for the children's group. The simulations indicated that the best fitting model per condition was able to capture learning on a trial-by-trial level in each age group (Supplementary Figure 4).

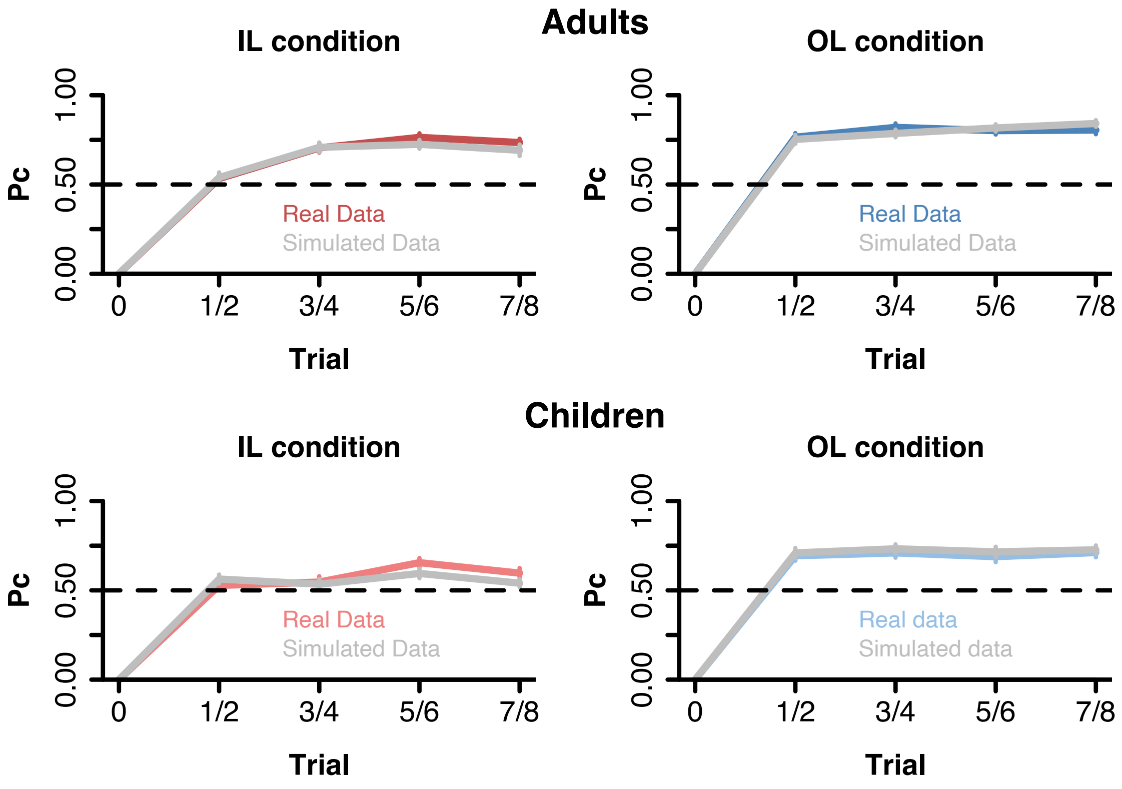

Supplementary Figure 7. **Plots of raw (Subject) and simulated data.** Plots show the average proportion of optimal choice for the raw (subject) data and simulated data per age group across trials. Simulated data are acquired using the best-fitting model per condition and the individual parameter values for each subject. The simulations are the result of the same number of trials (48)

and subjects per age group (i.e., for adults 30 iterations and for children 26 iterations). Shaded areas represent SEM.

(3) fMRI

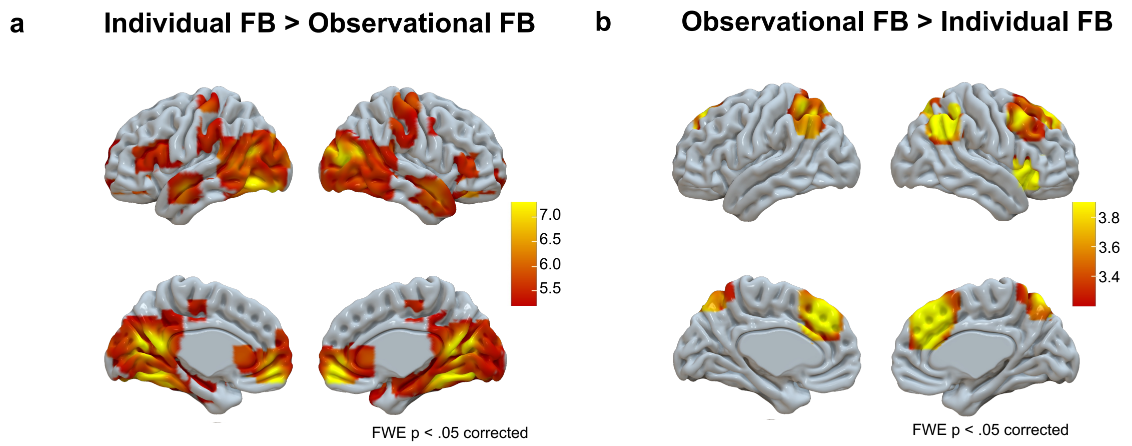

Supplementary Figure 8. **Activation clusters of feedback processing (unmodulated) comparing own outcomes in individual learning and other's outcomes in observational learning.** Whole-brain analyses for regions responding to **a** Individual > Observational feedback (FB) and **b** Observational > Individual feedback (FB). Displayed results are whole-brain FWE cluster-level corrected (*p*FWE < .05), with a primary voxel-wise threshold of $p < .001$.

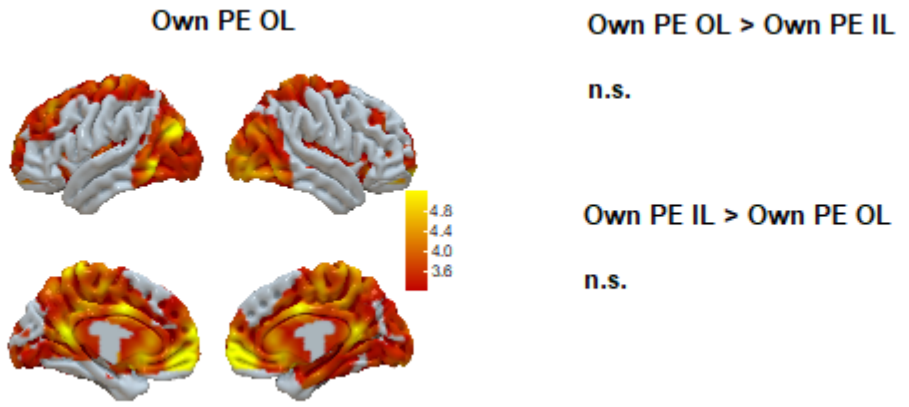

Supplementary Figure 9. **Activation clusters of PE activation (pos) during own outcomes in the observational learning (OL) condition.** When contrasting outcome-related PE activation in the OL and individual (IL) learning condition no significant differences were observed. Displayed results are whole-brain FWE cluster-level corrected (*p*FWE < .05), with a primary voxel-wise threshold of $p < .001$.

Supplementary References

1. Burke, C. J., Tobler, P. N., Baddeley, M. & Schultz, W. Neural mechanisms of observational learning. *Proc. Natl. Acad. Sci.* **107**, 14431–14436 (2010).
2. Sutton, R. S. & Barto, A. G. *Reinforcement learning: an introduction*. (MIT Press, 1998).
3. O'Doherty, J. P. Reward representations and reward-related learning in the human brain: insights from neuroimaging. *Curr. Opin. Neurobiol.* **14**, 769–776 (2004).
4. Team, R. C. R: A language and environment for statistical computing. (2013).
