# Supplementary material for: Observational reinforcement learning in children and young adults
Source: NPJ Sci Learn. 2024 Mar 13;9:18. doi: 10.1038/s41539-024-00227-9 (PMC10937639; doi:10.1038/s41539-024-00227-9)
Supplement: Supplementary file 2 — Reporting Summary [file 41539_2024_227_MOESM2_ESM.pdf]

nature portfolio

Corresponding author(s): Julia Rodriguez-Buritica
Anna van Duijvenvoorde

Last updated by author(s): Feb 7, 2024

Reporting Summary

Nature Portfolio wishes to improve the reproducibility of the work that we publish. This form provides structure for consistency and transparency in reporting. For further information on Nature Portfolio policies, see our Editorial Policies and the Editorial Policy Checklist.

Statistics

For all statistical analyses, confirm that the following items are present in the figure legend, table legend, main text, or Methods section.

n/a	Confirmed	
☐	☒	The exact sample size (n) for each experimental group/condition, given as a discrete number and unit of measurement
☐	☒	A statement on whether measurements were taken from distinct samples or whether the same sample was measured repeatedly
☐	☒	The statistical test(s) used AND whether they are one- or two-sided<br>*Only common tests should be described solely by name; describe more complex techniques in the Methods section.*
☐	☒	A description of all covariates tested
☐	☒	A description of any assumptions or corrections, such as tests of normality and adjustment for multiple comparisons
☐	☒	A full description of the statistical parameters including central tendency (e.g. means) or other basic estimates (e.g. regression coefficient) AND variation (e.g. standard deviation) or associated estimates of uncertainty (e.g. confidence intervals)
☐	☒	For null hypothesis testing, the test statistic (e.g. F, t, r) with confidence intervals, effect sizes, degrees of freedom and P value noted<br>*Give P values as exact values whenever suitable.*
☒	☐	For Bayesian analysis, information on the choice of priors and Markov chain Monte Carlo settings
☒	☐	For hierarchical and complex designs, identification of the appropriate level for tests and full reporting of outcomes
☐	☒	Estimates of effect sizes (e.g. Cohen's d, Pearson's r), indicating how they were calculated

Our web collection on statistics for biologists contains articles on many of the points above.

Software and code

Policy information about availability of computer code

Data collection	Behavioral data was collected with Eprime and paper/pencil (for exit-questionnaire). Functional MRI data were acquired with a standard whole-head coil using a 3-T Philips Achieva scanner
Data analysis	R, SPM8

For manuscripts utilizing custom algorithms or software that are central to the research but not yet described in published literature, software must be made available to editors and reviewers. We strongly encourage code deposition in a community repository (e.g. GitHub). See the Nature Portfolio guidelines for submitting code & software for further information.

Data

Policy information about availability of data

All manuscripts must include a data availability statement. This statement should provide the following information, where applicable:

- Accession codes, unique identifiers, or web links for publicly available datasets
- A description of any restrictions on data availability
- For clinical datasets or third party data, please ensure that the statement adheres to our policy

The data that supports findings of this study will be made available in the Leiden repository: https://openaccess.leidenuniv.nl

Human research participants

Policy information about studies involving human research participants and Sex and Gender in Research.

Reporting on sex and gender	Sex- and gender-based analyses were not the main focus of our paper and due to limited sample sizes per age group we can not further perform such analyses. We report the number of males/females in our sample.
Population characteristics	The final sample consisted of 30 adults (Age Mean (SD) = 19.45 (.86); 16 female) and 29 children (Age Mean (SD) = 9.71 (.89); 18 female).
Recruitment	Participants were recruited through local advertisements
Ethics oversight	Ethics Committee of the Leiden University Medical Center

Note that full information on the approval of the study protocol must also be provided in the manuscript.

Field-specific reporting

Please select the one below that is the best fit for your research. If you are not sure, read the appropriate sections before making your selection.

☐ Life sciences ☒ Behavioural & social sciences ☐ Ecological, evolutionary & environmental sciences

For a reference copy of the document with all sections, see nature.com/documents/nr-reporting-summary-flat.pdf

Behavioural & social sciences study design

All studies must disclose on these points even when the disclosure is negative.

Study description	Quantitative experimental cross-sectional fMRI-study
Research sample	Participants (children and young adults) were recruited through local advertisements and existing databases. Participants received a compensation for their time
Sampling strategy	Sample size was determined based on an intendend N per age group with a minimum of N=30. We approached participants through existing databases and local advertisements that fell into the pre-determined age categories (children ages 8-10; young adults ages 18-25). We tried ot balance the number of males/females in both age groups.
Data collection	We used computer-recorded behavioral responses, and brain-based funcitonal imaging data. This data was collected in the MR scanner. Exit-questionnaires were recorded data via pen and paper after the experiment. The researchers were ot blind to the experimental conditions and the study hypothesis
Timing	Start of data collection was April 2016. End of data collection was end of June 2016
Data exclusions	Data of one child was excluded due to inability to complete the task. For occasional occurring head motion (Framewise displacements > .5) volumes with motion were flagged and were not included in regressors of interest but instead modeled by nuisance regressors (i.e., censored), number of censored volumes regressors varied between 2-12 (<10% of volumes).
Non-participation	One child did not complete the imaging task. No other participants dropeed out of the study or did not finish the study protocol.
Randomization	Participants were not allocated to experimental groups.

Reporting for specific materials, systems and methods

We require information from authors about some types of materials, experimental systems and methods used in many studies. Here, indicate whether each material, system or method listed is relevant to your study. If you are not sure if a list item applies to your research, read the appropriate section before selecting a response.

Materials & experimental systems

n/a	Involved in the study
☒	☐ Antibodies
☒	☐ Eukaryotic cell lines
☒	☐ Palaeontology and archaeology
☒	☐ Animals and other organisms
☒	☐ Clinical data
☒	☐ Dual use research of concern

Methods

n/a	Involved in the study
☒	☐ ChIP-seq
☒	☐ Flow cytometry
☐	☒ MRI-based neuroimaging

Magnetic resonance imaging

Experimental design

Design type

Event-related design

Design specifications

3 blocks á 32 trials, duration of the experiment around 30 min

Behavioral performance measures

correct choice, response time

Acquisition

Imaging type(s)

functional, structural

Field strength

3-T

Sequence & imaging parameters

T2*-weighted echoplanar images (EPIs) were obtained during three functional runs, in which the first two volumes were discarded to allow for equilibration of T1 saturation effects. Volumes covered the whole brain (38 slices; 2.75 mm slice thickness; ascending acquisition) and were acquired every 2200 ms (TE = 30 ms). A high resolution T1-weighted anatomical scan was included at the end of the imaging protocol (140 slices; TR=9.76 ms; TE=4.59 ms; flip angle=8°; FOV=224×177.33×168 mm; in-plane resolution=0.875x0.875 mm; slice thickness=2 mm). Visual stimuli were projected onto a screen that was visible for participants via a mirror attached to the head coil.

Area of acquisition

Whole brain scans

Diffusion MRI ☐ Used ☒ Not used

Preprocessing

Preprocessing software

Data preprocessing and analysis were conducted using SPM8 (Welcome Department of Cognitive Neurology, London). Images were corrected for differences in timing of slice acquisition, followed by rigid body motion correction. Functional volumes were smoothed with a 6-mm full-width at half maximum isotropic Gaussian kernel.

Normalization

The normalization algorithm used a 12-parameter affine transformation together with a nonlinear transformation involving cosine basis functions. During normalization the data was re-sampled to 3-mm cubic voxels.

Normalization template

Templates were based on the MNI305 stereotaxic space

Noise and artifact removal

Trials in which participants did not respond on time and censored motion trials were modeled separately as regressors of no interest. Finally, 6 head-motion parameters were included as nuisance regressors.

Volume censoring

For occasional occurring head motion (Framewise displacements > .5) volumes with motion were flagged and were not included in regressors of interest but instead modeled by nuisance regressors (i.e., censored), number of censored volumes regressors varied between 2-12 (<10% of volumes).

Statistical modeling & inference

Model type and settings

To investigate the neural responses to own and other's outcomes and prediction errors, we modeled in separate regressors the onset of the choice stimuli with the reaction time as the duration in the observational and the action phase for both conditions. Choice value, derived from the reinforcement learning model, was included as a parametric modulator of the choice regressor in the observational (OL), and of the choice regressor in the action phase (OL, IL). The onset of the outcome was modeled with a stick function. Separate outcome regressors were created for own and other's outcomes in the observational condition, and for own and no-outcomes in the individual condition. In addition, three outcome regressors (own and other's outcomes in the observational, and own outcomes in the individual condition) included a parametric modulation of trial-wise prediction errors derived from the reinforcement learning model.

Effect(s) tested

Our main analyses include the comparison between own outcomes in the IL (action phase), and other's outcomes in the OL condition (observational phase, see Figure 1), and whether this interacted with age group (children, adults). In addition, we

test age group effects on IL (action phase) and OL (observational phase) separately. For completeness we include whole-brain maps of the non-modulated feedback event in Figure S6.

Specify type of analysis: ☒ Whole brain ☐ ROI-based ☐ Both

Statistic type for inference (See Eklund et al. 2016)	Unless stated otherwise, task-related responses were considered significant if they exceeded an FWE cluster-corrected threshold of $p < .05$, with an initial threshold of $p < .001$
Correction	Unless stated otherwise, task-related responses were considered significant if they exceeded an FWE cluster-corrected threshold of $p < .05$, with an initial threshold of $p < .001$.

Models & analysis

n/a	Involved in the study
☒	☐ Functional and/or effective connectivity
☒	☐ Graph analysis
☒	☐ Multivariate modeling or predictive analysis
